# Acute lymphoblastic leukemia displays a distinct highly methylated genome

**DOI:** 10.1038/s43018-022-00370-5

**Published:** 2022-05-19

**Authors:** Sara Hetzel, Alexandra L. Mattei, Helene Kretzmer, Chunxu Qu, Xiang Chen, Yiping Fan, Gang Wu, Kathryn G. Roberts, Selina Luger, Mark Litzow, Jacob Rowe, Elisabeth Paietta, Wendy Stock, Elaine R. Mardis, Richard K. Wilson, James R. Downing, Charles G. Mullighan, Alexander Meissner

**Affiliations:** 1 Department of Genome Regulation, Max Planck Institute for Molecular Genetics, Berlin, Germany; 2 Department of Stem Cell and Regenerative Biology, Harvard University, Cambridge, MA USA; 3 Department of Molecular and Cellular Biology, Harvard University, Cambridge, MA USA; 4 Department of Pathology, St Jude Children’s Research Hospital, Memphis, TN USA; 5 Department of Computational Biology, St Jude Children’s Research Hospital, Memphis, TN USA; 6 Center for Applied Bioinformatics, St Jude Children’s Research Hospital, Memphis, TN USA; 7 Abramson Cancer Center, University of Pennsylvania, Philadelphia, PA USA; 8 Division of Hematology, Department of Internal Medicine, Mayo Clinic, Rochester, MN USA; 9 Department of Hematology, Shaare Zedek Medical Center, Jerusalem, Israel; 10 Department of Oncology, Montefiore Medical Center, Bronx, NY USA; 11 University of Chicago Comprehensive Cancer Center, Chicago, IL USA; 12 Institute for Genomic Medicine, Nationwide Children’s Hospital, Columbus, OH USA; 13 Broad Institute of MIT and Harvard, Cambridge, MA USA; 14 Department of Biology, Chemistry and Pharmacy, Freie Universität, Berlin, Germany

**Keywords:** Haematological cancer, Cancer genomics, Cancer epigenetics, Cancer

## Abstract

DNA methylation is tightly regulated during development and is stably maintained in healthy cells. In contrast, cancer cells are commonly characterized by a global loss of DNA methylation co-occurring with CpG island hypermethylation. In acute lymphoblastic leukemia (ALL), the commonest childhood cancer, perturbations of CpG methylation have been reported to be associated with genetic disease subtype and outcome, but data from large cohorts at a genome-wide scale are lacking. Here, we performed whole-genome bisulfite sequencing across ALL subtypes, leukemia cell lines and healthy hematopoietic cells, and show that unlike most cancers, ALL samples exhibit CpG island hypermethylation but minimal global loss of methylation. This was most pronounced in T cell ALL and accompanied by an exceptionally broad range of hypermethylation of CpG islands between patients, which is influenced by TET2 and DNMT3B. These findings demonstrate that ALL is characterized by an unusually highly methylated genome and provide further insights into the non-canonical regulation of methylation in cancer.

## Abstract (web summary)

Using genome-wide bisulfite sequencing of acute lymphoblastic leukemia subtypes, cell lines and healthy cells, Hetzel et. al. find that unlike most cancers, ALL has a highly methylated genome, which points to a distinct mode of epigenome regulation in this cancer type.

## Main

The DNA methylation landscape of somatic cells follows a bimodal pattern where the majority of the genome is highly methylated while CpG-dense regions, termed CpG islands (CGIs), and clusters of CGIs defined as DNA methylation valleys (DMVs) or canyons, usually remain free of methylation^1^. These patterns frequently shift toward a globally hypomethylated genome during tumorigenesis accompanied by hypermethylation targeted to thousands of CGIs and hundreds of DMVs^2–7^. The loss of methylation can affect the whole genome but is most pronounced in so-called partially methylated domains (PMDs), megabase-scale regions that coincide with late-replication timing and low density of both CpGs and genes^8,9^. Within the PMDs, isolated CpGs are most affected and for these a notable decrease occurs in extraembryonic lineages, aging and tumorigenesis^9^. The precise cause and consequence are not known, but have been hypothesized to be the result of inefficient DNA re-methylation after replication^9^ and more recently suggested to act as a self-defense mechanism of the cell^10^.

Aberrant expression and mutant variants of epigenetic regulators such as de novo DNA methyltransferases (DNMT3A and DNMT3B) and ten-eleven translocation (TET) enzymes have been observed in a few cancers and might therefore contribute to specific changes of the methylome. Loss-of-function mutations in DNMT3A lead to a focal loss of methylation^11^, whereas mutations in IDH1/2 enzymes lead to oncometabolite production that inhibits the TETs and leads to increased hypermethylation of CGIs^12^. *DNMT3A* and *TET2* mutations are most frequently described in hematopoietic malignancies of the myeloid lineage^13–15^. However, despite many commonly shared alterations to the DNA methylation landscape across cancer types, none of these mutations are frequent across indications.

ALL is the most common pediatric cancer and consists of multiple subtypes with distinct gene expression profiles defined by constellations of somatic mutations, chromosomal rearrangements deregulating oncogenes or encoding chimeric fusion transcripts and aneuploidy^16–20^. Furthermore, aberrant DNA methylation has also been used to characterize established subtypes and stratify risk groups of patients with ALL^21^. However, previous studies examining DNA methylation in ALL mostly utilized selective enrichment strategies as well as array-based approaches that prioritize CGIs and coding regions and therefore lack representation of the complete genome^22–27^. In contrast, the few limited genome-wide sequencing-based studies of ALL subtypes have focused on specific subtypes, such as *ETV6-RUNX1*, high hyperdiploidy, unclassified B cell ALL (B-ALL) and T cell ALL (T-ALL) and reported contradicting results ranging from a mild increase in global DNA methylation^28^ to significant hypomethylation^29,30^.

In T-ALL, a subset of patients has been shown to exhibit a CpG island methylator phenotype (CIMP) coinciding with higher expression of ANTP homeobox genes, shorter telomere length and higher mitotic age, which implicates potentially different routes of tumorigenesis compared to non-CIMP T-ALL cases^31^. Notably, patients who are CIMP-positive have been reported to have a better prognosis and survival rate^31^. Using mouse models of T-ALL, it has been suggested that a preleukemic phase could lead to the establishment of CIMP and is responsible for the increased mitotic age associated with increased CGI hypermethylation^32^. So far, no direct correlation between CIMP subtypes and mutations in epigenetic regulators or their expression have been identified^31^. Expression level changes of key DNA methylation-related enzymes in T-ALL orchestrated by MYC have been described based on observations in mouse models and T-ALL cancer cell lines; however, without linking these changes to a potential CGI-related hypermethylation phenotype in patients with T-ALL^33^. Further investigation is needed to connect these observations to T-ALL methylation in patients and associated CIMP subtypes.

In this study, using integrated genomic analysis of a large cohort of B-progenitor and T-lineage ALL, corresponding cell lines and healthy samples, we describe the distinct methylome of ALL and provide insights into the epigenomic alterations in leukemogenesis.

## Results

### Genome-wide methylation maps of acute lymphoblastic leukemia

**Fig. 1:**
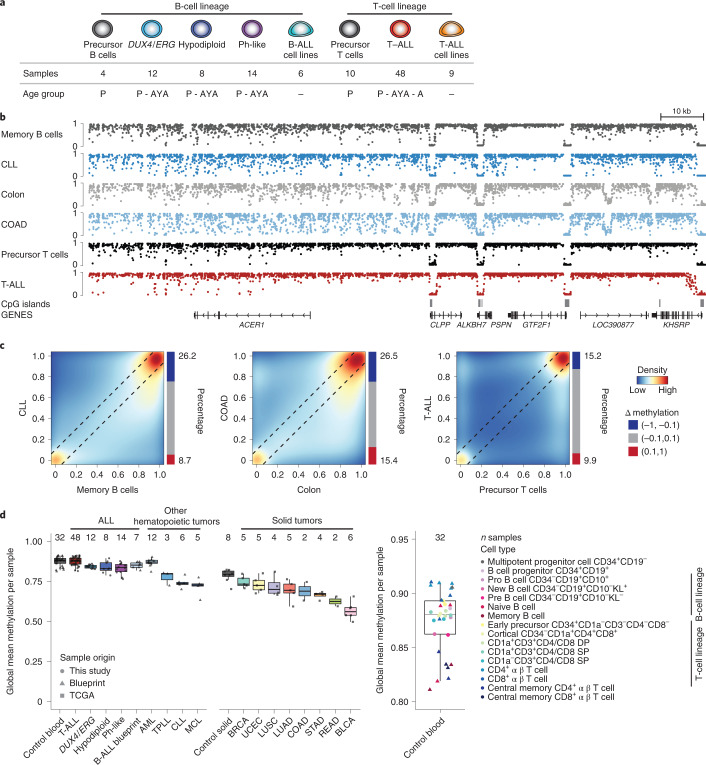
The ALL genome is unusually highly methylated. **a**, Cohort overview including all examined ALL subtypes with age information (P, pediatric aged 0–15 years; AYA, adolescents and young adults aged 16–39 years; A, adults aged ≥40 years). Healthy cells were purified by fluorescence-activated cell sorting from bone marrow of children without leukemia and neonatal thymi. **b**, Genome browser tracks for WGBS data of representative memory B cells, healthy colon tissue, precursor T cells, chronic lymphocytic leukemia (CLL), colon adenocarcinoma (COAD) and T-ALL for an exemplary locus (*ACER1* and neighboring genes; chr19:6,282,123–6,425,048). CLL and COAD display the characteristic global loss of methylation in comparison to their respective healthy tissue, whereas T-ALL methylation remains more highly methylated and comparable to precursor T cells. **c**, Correlation of CpG methylation levels between memory B cells and CLL, healthy colon and COAD as well as precursor T cells and T-ALL (blue, low density; red, high density, same samples as in **b**). Black lines mark the difference of 0.1 from the diagonal in both directions. **d**, Global methylation levels averaged across all covered CpGs outside of CGIs (CpGs in CGIs are excluded to not bias global quantifications by potential differences in CpG-dense regions) per sample for ALL subtypes, other hematopoietic malignancies from Blueprint and solid tumors from TCGA (left) as well as healthy cell types of the lymphoid lineages from this study and Blueprint (right) (Supplementary Tables 2 and 3). Lines denote the median, edges denote the interquartile range (IQR), whiskers denote 1.5 × IQR and minima/maxima are represented by dots. The number of independent samples is indicated at the top. Blueprint cancer types include acute myeloid leukemia (AML), T-cell prolymphocytic leukemia (TPLL), CLL and mantle cell lymphoma (MCL). TCGA tumor types include bladder urothelial carcinoma (BLCA), breast invasive carcinoma (BRCA), COAD, lung adenocarcinoma (LUAD), lung squamous cell carcinoma (LUSC), rectum adenocarcinoma (READ), stomach adenocarcinoma (STAD) and uterine corpus endometrial carcinoma (UCEC). T-ALL and AML show global DNA methylation levels comparable to hematopoietic control cells, whereas B-ALL subtypes show a mild loss not comparable to the drastic loss of other tumor types. DP, double positive; SP, single positive.

We performed whole-genome bisulfite sequencing (WGBS) on diagnostic leukemic cells from 82 patients representing three subtypes of childhood B-ALL (Philadelphia chromosome (Ph)-like^34^, *DUX4*-rearranged/*ERG*-deregulated^19^ and hypodiploid^20^ ALL) and T-ALL. The T-ALL cases included pediatric (*n* = 30), adolescents (*n* = 16) and adults (*n* = 1). A large selection of samples was also previously subjected to whole-genome and transcriptome sequencing (Supplementary Table 1). In addition, we generated data for five B-ALL and nine T-ALL cell lines representing these ALL subtypes as well as healthy B- and T-cell progenitor populations (Fig. 1a and Supplementary Table 1). In total we paired-end sequenced ~60 billion fragments (~536 million fragments on average per sample), which resulted in ~22 million CpGs at 10× coverage per sample across autosomes (Supplementary Table 2). A key advantage of these deep-sequencing data is that more CpGs are consistently covered across samples, retaining approximately 20 million CpGs captured across 80% of healthy and tumor samples.

### ALL displays an unusually highly methylated genome

**Extended Data Fig. 1:**
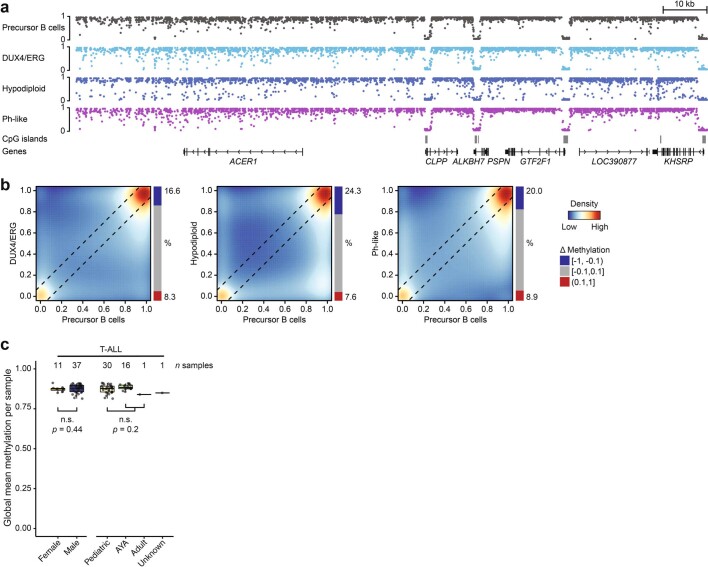
Limited loss of methylation in B-ALL. **a)** Genome browser tracks for WGBS data of representative precursor B cells and B-ALL subtypes for an exemplary locus (*ACER1* and neighboring genes; chr19:6,282,123-6,425,048, same as in Fig. 1b). B-ALL subtypes exhibit only mild loss of global methylation compared to their healthy counterpart. **b)** Correlation of CpG methylation levels between precursor B cells and B-ALL subtypes (blue = low density, red = high density, same samples as in Fig. 1b). Black lines mark the difference of 0.1 from the diagonal in both directions. **c)** Global methylation levels averaged across all covered CpGs outside of CGIs per sample for T-ALL samples split by sex and age. Lines denote the median, edges denote the IQR, whiskers denote 1.5 × IQR and minima/maxima are represented by dots. The number of independent samples is indicated at the top. No significant differences were detected between the groups (two-sided Wilcoxon rank-sum test).

Global loss of methylation has long been viewed as a characteristic feature accompanying tumorigenesis^35,36^, which is readily seen in representative WGBS data of chronic lymphocytic leukemia (CLL) and colon cancer (COAD) (Fig. 1b,c). In contrast, T-ALL exhibited a global DNA methylation landscape comparable to precursor T cells derived from healthy infant thymi, whereas B-ALL samples showed mild loss of methylation at varying degrees (Fig. 1b,c and Extended Data Fig. 1a,b). We next extended these analyses to additional hematopoietic and solid tumors (Fig. 1d and Supplementary Table 3), which again highlights the unusual high methylation levels in T-ALL as well as in acute myeloid leukemia (AML)^9^. Age and sex did not affect this unexpected aspect of the T-ALL cancer methylome (Extended Data Fig. 1c; *P* = 0.2 and *P* = 0.44, respectively, Wilcoxon rank-sum test). B-ALL samples also remained more highly methylated but showed minor loss of methylation, which was most pronounced for Ph-like ALL (Fig. 1d). Similar observations were made for a limited set of pediatric B-ALL samples of unknown subtypes (Blueprint)^9^.

### Partially methylated domains remain highly methylated in T-ALL

**Fig. 2:**
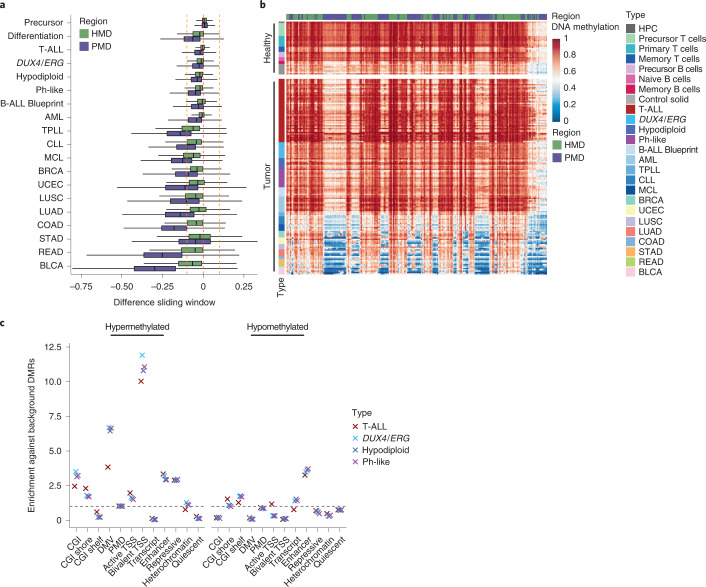
Partially methylated domains remain highly methylated in ALL. **a**, Distribution of methylation differences across frequently covered sliding windows based on subtype averages split by HMDs and PMDs (*n* = 3,228,539 and 3,729,433 windows, respectively; 1 kb size, 250-bp steps across the genome, excluding CGIs, covered by at least 80% of subtypes considered). Lines denote the median, edges denote the IQR and whiskers denote either 1.5 × IQR or minima/maxima (if no point exceeded 1.5 × IQR; outliers were omitted). Each tumor type is compared to its respective healthy tissue. The difference between precursor T and B cells (precursor control, first) is shown as measure for healthy, same-stage cell-type-specific differences. The difference between memory and precursor B cells (differentiation control, second) is shown as measure for natural differences occurring during B-cell differentiation. T-ALL shows the smallest differences between averaged tumor and control samples, which slightly increases for B-ALL subtypes. The loss in ALL in general, however, is less pronounced than the loss during B-cell differentiation. **b**, Average methylation of solo-WCGW CpGs across PMDs (blue) and HMDs (green) for an exemplary locus (chromosome 16p) for a range of healthy tissues, ALL subtypes, other hematopoietic malignancies and solid tumors. **c**, Enrichment of DMRs against random background DMRs in different features (>1, enrichment; 1, no difference (represented by the dashed line); <1, depletion). T-ALL and B-ALL subtypes show enrichment in similar features but to different extents. DMRs were called per subtype against the respective control cell type (*n* samples as shown in Fig. 1a). The number of DMRs per subtype are shown in Extended Data Fig. 2c. The number of random background DMRs per subtype equals 1,000 times the number of DMRs per subtype (Methods).

**Extended Data Fig. 2:**
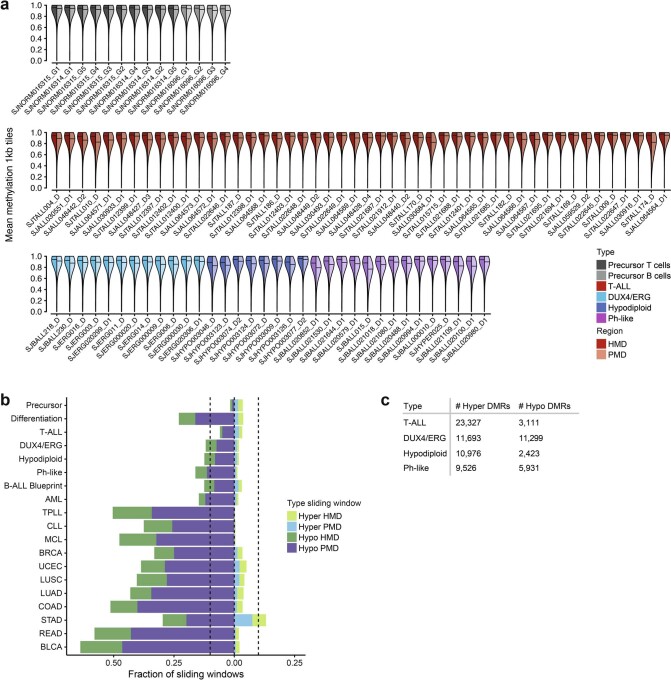
Patient samples display consistently high methylation. **a)** Violin plots showing the distribution of methylation in 1-kb tiles separated into HMDs and PMDs (*n* = 650,158 and 597,186 tiles respectively) for every healthy and ALL sample within our cohort. Lines denote the median. **b)** Fraction of sliding windows that are hyper- or hypomethylated in HMDs or PMDs per subtype comparison (difference > 0.1 or < -0.1 respectively). **c)** Number of hyper- and hypomethylated DMRs per ALL subtype.

In all cancer types that show global loss of methylation it preferentially accumulates in PMDs and is most pronounced at CpGs not flanked by other CpG sites (termed solo-WCGW CpGs)^9^. To further characterize the absence of global hypomethylation in T-ALL, using a sliding window approach we measured the loss of methylation in highly methylated domains (HMDs) and PMDs, excluding CpGs located in CGIs for ALL subtypes as well as other hematopoietic and solid tumors. We observed that on average T-ALL samples did not deviate much from the average precursor T-cell controls (Fig. 2a and Extended Data Fig. 2a,b). Minor shifts toward hypomethylation were found for B-ALL subtypes and AML, always slightly more pronounced in PMDs compared to HMDs. However, the modest genome-wide loss of methylation in both B-ALL and AML was less pronounced than the genome-wide loss that occurs during healthy B-cell differentiation (calculated by comparing memory to early precursor B cells; Fig. 2a). Other hematopoietic as well as solid tumors exhibited more pronounced hypomethylation in HMDs as well as PMDs, with some tumor types showing extreme loss of methylation in PMDs (rectum adenocarcinoma (READ) and bladder urothelial carcinoma (BLCA)). When additionally analyzing solo-WCGW CpG methylation in PMDs across chromosome 16p, as presented previously^9^, we observed that these CpGs, which are generally most prone to loss of methylation, were not strongly hypomethylated in T-ALL compared to healthy precursor T cells, in sharp contrast to solid tumors and most other hematopoietic cancer types (Fig. 2b). Previously, it has been hypothesized that a lack of hypomethylation in pediatric tumors might stem from the generally higher methylation levels in cells from younger patients^9^; however, adolescent and adult T-ALL samples in our cohort also lacked a significant reduction of methylation in PMDs (*P* = 0.21, Wilcoxon rank-sum test, pediatric versus AYA/adult T-ALL).

### T- and B-ALL subtypes share features of the altered methylome

We next examined local DNA methylation changes for each ALL subtype by calling differentially methylated regions (DMRs) to identify sites with a significant difference across samples compared to their respective non-tumor control (DMR mean absolute difference >0.2, *q* value <0.05). We compared T-ALL samples with precursor T cells and *DUX4*/*ERG*-rearranged, hypodiploid and Ph-like B-ALL samples with precursor B cells, respectively (Supplementary Tables 4–7). T-ALL showed a high number of hypermethylated (88% of all subtype-specific DMRs) and only a few hypomethylated DMRs, whereas B-ALL subtypes showed more hypomethylated DMRs (Extended Data Fig. 2c). Hypermethylated DMRs were enriched across all ALL subtypes in CpG-dense genomic features (CGIs and DMVs) as well as H3K27me3-marked chromatin states predicted for hematopoietic stem cells (HSCs) when compared to random control regions (Fig. 2c). These results confirm the absence of strong hypomethylation in T-ALL also at the local level and indicate that DNA methylation changes across B- and T-ALL affect similar types of regions.

### Global methylation levels correlate with CGI hypermethylation

**Fig. 3:**
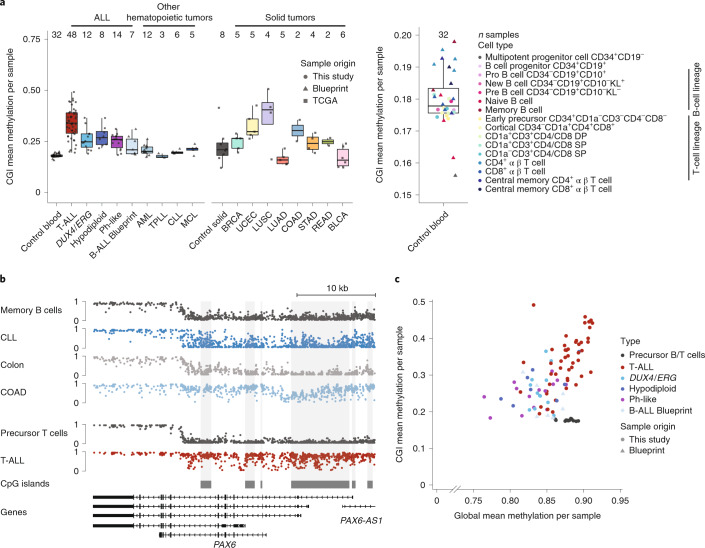
Global and CGI methylation are correlated across ALL subtypes. **a**, CGI methylation levels averaged across all covered CpGs per sample for ALL subtypes, other hematopoietic malignancies from Blueprint and solid tumors from TCGA (left) as well as healthy cell types of the lymphoid lineages from this study and Blueprint (right) (Supplementary Tables 2 and 3). Lines denote the median, edges denote the IQR, whiskers denote 1.5 × IQR and minima/maxima are represented by dots. The number of independent samples is indicated at the top and is the same as in Fig. 1d. All tumor types exhibit CGI hypermethylation to a varying degree with the largest range visible for T-ALL. **b**, Genome browser tracks for WGBS data of representative memory B and precursor T cells, healthy colon tissue, CLL, COAD and T-ALL for an exemplary locus (*PAX6* gene; chr11:31,806,145–31,844,510). Cancer samples show CGI hypermethylation in comparison to their respective healthy tissue to different extents. **c**, Correlation between global mean methylation (excluding CpGs in CGIs) and CGI mean methylation levels across ALL subtypes and healthy control samples. For all ALL subtypes, most prominent for T-ALL, a correlation between global and CGI methylation levels can be observed. The number of samples per ALL subtype is the same as in **a**.

**Extended Data Fig. 3:**
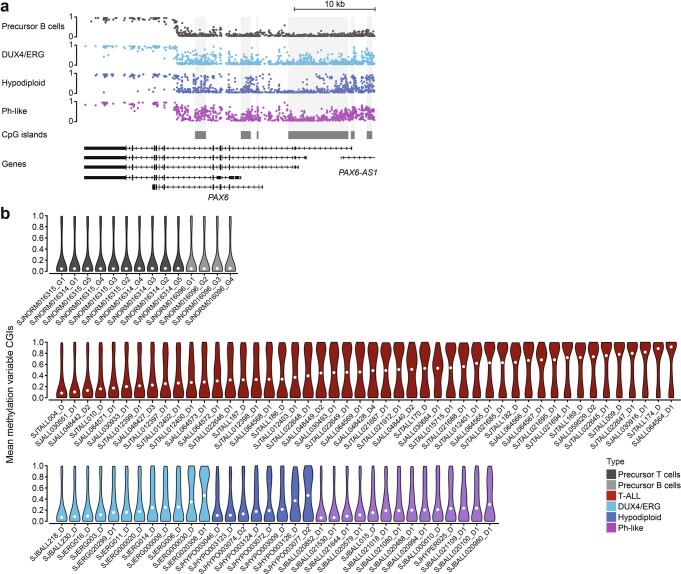
CGI hypermethylation levels show a broad range across T-ALL samples. **a)** Genome browser tracks for WGBS data of representative precursor B cells and B-ALL subtypes for an exemplary locus (*PAX6* gene; chr11:31,806,145-31,844,510, same as in Fig. 3b). Cancer samples show CGI hypermethylation in comparison to their respective healthy tissue to different extents. **b)** Violin plots showing the distribution of methylation in variably methylated CGIs (*n* = 9,349, determined based on all healthy and ALL samples) for every healthy and ALL sample within our cohort. White dots denote the median.

Similar to most cancer types, hypermethylated DMRs showed an enrichment in CGIs across ALL subtypes. We therefore aimed to investigate the nature of this CGI hypermethylation with respect to both other hematopoietic and solid tumor types (Fig. 3a,b and Extended Data Fig. 3a,b). When comparing T-ALL with CLL and COAD, all three examples show CGI hypermethylation compared to their respective healthy sample although the specific local architecture across a given CGI is distinct for each (Fig. 3b). T-ALLs were notable for their higher levels of CGI methylation and larger inter-sample variation, even in a pan-cancer comparison (Fig. 3a and Supplementary Table 3). Notably, a comparison of global average methylation with average methylation levels of CGIs across patients with ALL revealed a positive trend, especially for the T-ALL samples. The average CpG methylation within CGIs ranged from 0.2, corresponding to the lowest genome-wide average methylation, to almost 0.5 for samples with the highest genome-wide average methylation (Fig. 3c).

### T-ALL patients exhibit a wide range of hypermethylation levels

**Fig. 4:**
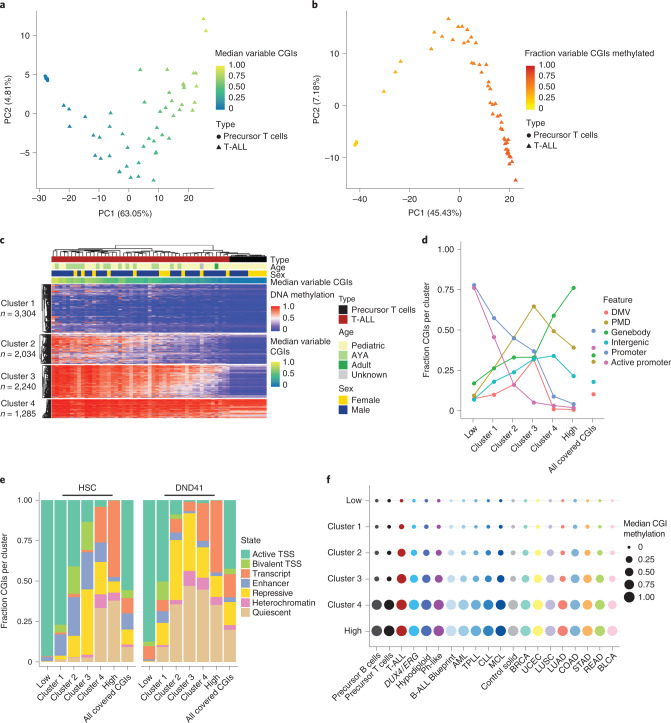
Methylation levels in T-ALL define different clusters of CGIs. **a**, PCA based on the mean methylation of the variable commonly covered CGIs (*n* = 8,863 CGIs) of precursor T cells and patients with T-ALL. T-ALL samples show continuous CGI methylation levels instead of forming groups of high and low CGI methylation (*n* = 10 precursor T cell and 48 T-ALL samples). **b**, PCA based on the methylation status of the variable commonly covered CGIs (methylated defined as >0.2) of precursor T cells and patients with T-ALL (*n* samples and CGIs same as in **a**). **c**, Hierarchical clustering representing four clusters of CGIs identified by consensus clustering of variable CGIs across patients with T-ALL and healthy precursor T cells. Cluster 1 shows already a range from low to high hypermethylation across patients with T-ALL although targets are still rather sample-specific. Cluster 2 and 3 show dynamic, increasing hypermethylation across patients. CGIs in cluster 1 to 3 are unmethylated in precursor T cells, whereas cluster 4 shows higher methylation levels that become fully methylated in almost all patients with T-ALL. **d**, Fraction of CGIs per cluster overlapping promoters, active promoters (defined for genes with an average transcript per million (TPM) ≥1 across different precursor T-cell stages), DMVs, PMDs, gene bodies and intergenic regions. The fraction of CGIs in promoters decreases with increasing cluster-wise methylation level, whereas the proportion overlapping gene bodies, PMDs and intergenic regions rises (defined as 20% of a CGI or 20% of a feature overlapping). **e**, Fraction of CGIs per cluster overlapping chromatin states in HSCs and the T-ALL cell line DND41 (as a proxy for T-ALL). A CGI was assigned to the chromatin state with the largest overlap. **f**, Median methylation per CGI cluster for ALL subtypes, other hematopoietic malignancies and solid tumors. Although defined based on T-ALL, the clusters of CGIs show a similar tendency to gain methylation from cluster 1 to 4 as well as for the low/high group in other cancer types. Samples per tumor type (*n* same as in Fig. 1d) were averaged to generate a subtype-specific methylation signature (Methods).

**Extended Data Fig. 4:**
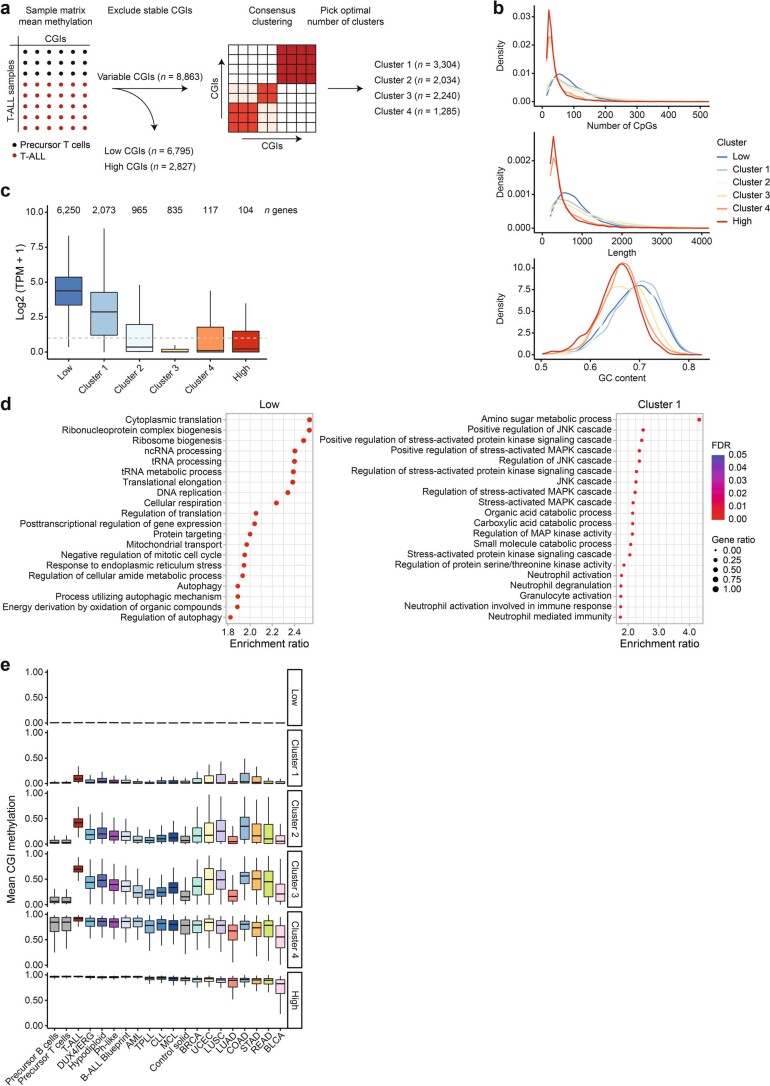
Association of CGI clusters with genomic and transcriptomic features. **a)** Schematic of CGI clustering: Commonly covered CGIs across precursor T cells and T-ALL patients were used. Stably highly or lowly methylated CGIs across all samples were excluded from the clustering. The remaining variably methylated CGIs were clustered based on their average methylation across the samples using consensus clustering with partitioning around medoids (PAM) distance measure and 100 repetitions yielding four clusters of CGIs. **b)** Density of CGI characteristics per cluster: Number of CpGs (top), length in base pairs (middle) and GC content (bottom). Consistently lowly methylated CGIs and cluster 1 exhibit the highest number of CpGs, GC content and length compared to other, more methylated clusters. **c)** Log2-transformed expression of genes associated with a promoter CGI per CGI cluster in healthy precursor T cells. Lines denote the median, edges denote the IQR and whiskers denote either 1.5 × IQR or minima/maxima (if no point exceeded 1.5 × IQR; outliers were omitted). The number of genes per boxplot is indicated at the top. Genes associated with the low group/cluster 1 are mostly expressed while genes associated with the remaining clusters are mostly already silenced in precursor T cells indicating that more methylation at their promoters will in many cases not influence their expression status. **d)** Overrepresentation analysis of genes associated with promoter CGIs of the low group and cluster 1 in biological processes. Genes with a promoter CGI of the low group are mainly involved in cell maintenance functions while genes with a promoter CGI belonging to cluster 1 show enrichment in the MAPK and JNK cascades. **e)** Boxplot of CGIs across all CGI clusters for ALL subtypes, other hematopoietic malignancies and solid tumors (low (*n* = 6,795), cluster 1 (*n* = 3,304), cluster 2 (*n* = 2,034), cluster 3 (*n* = 2,240), cluster 4 (*n* = 1,285), high (*n* = 2,827), samples per subtypes were averaged per CGI). Lines denote the median, edges denote the IQR and whiskers denote either 1.5 × IQR or minima/maxima (if no point exceeded 1.5 × IQR; outliers were omitted). Despite being defined on T-ALL subtypes, the trend of increasing methylation from low to high can be observed in B-ALL and other tumors as well.

Previous studies reported a CIMP subtyping of patients with T-ALL^31^. To inspect this further, we focused on commonly covered, variable CGIs (*n* = 8,863, not below 0.2 or above 0.8 in all control and T-ALL samples) and used principal component analysis (PCA) of precursor T cells and T-ALL samples based on the average methylation per CGI (Fig. 4a). This indicated that rather than a clear separation of specific methylation-based groups, patient samples instead distribute by their median CGI methylation and hence display a rather continuous range from levels close to healthy precursor T cells (median = 0.03) to more extreme hypermethylation (median = 0.88). PCA using the methylation status of variable CGIs per sample (methylation defined as > 0.2) showed a similar trend suggesting that not only CGI methylation levels rise continuously across samples but also the number of methylated CGIs (Fig. 4b). To further characterize the difference in hypermethylation level and targets across T-ALL subtypes, we performed a consensus clustering of the variable CGIs (Fig. 4c, Extended Data Fig. 4a and Supplementary Table 8) and identified four clusters of CGIs; cluster 1 consists of generally unmethylated CGIs in control samples with a sporadic/gradual gain of methylation across T-All samples. The group of unmethylated CGIs and cluster 1 show the highest GC content, number of CpGs within the island and largest size (Extended Data Fig. 4b)^37^. Cluster 2 and 3 also consist of mostly unmethylated CGIs in control samples. However, methylation increases from low to highly methylated T-ALL samples, where cluster 2 shows rather heterogeneous, sample-specific effects and cluster 3 a relatively homogeneous gain across CGIs for all patients. T-ALL samples with overall high CGI methylation levels reached up to 100% of methylation. Cluster 4 includes CGIs that were also methylated in control samples and become fully methylated in almost all samples (Fig. 4c).

### Chromatin state is linked to CGI methylation levels in T-ALL

To further characterize the T-ALL CGI clusters, we analyzed the overlap of CGIs per cluster, including the stably low and highly methylated CGIs (group low and high) with methylation- and gene-based features (Fig. 4d). Many lowly methylated CGIs (unmethylated group and cluster 1) are located in promoters and outside of gene bodies, intergenic regions or PMDs. The fraction of CGIs in promoters decreased along with the elevated methylation across clusters (cluster 2 to 4, methylated clusters), whereas the fraction of CGIs overlapping gene bodies, PMDs and intergenic regions increased. This agrees with previous findings that showed preferential CGI hypermethylation within PMDs^38^. More than 75% of CGIs in the group of consistently highly methylated CGIs overlap gene bodies, which is presumably linked to their transcriptional activity in healthy and tumor cells^39–42^ (Fig. 4d). Next, we calculated the fraction of CGIs per cluster overlapping specific chromatin states in HSCs as well as the cell line DND41 (Fig. 4e). As observed in Fig. 4d, the proportion of active and bivalent transcription start sites (TSSs) in HSCs decreased from cluster 1 to cluster 4. The proportion of CGIs overlapping Polycomb-repressed, heterochromatin and quiescent regions in HSCs increased from mainly unmethylated to methylated. In line with these findings, genes associated with cluster 1 or the low group correspond to actively transcribed genes in precursor T cells, whereas genes associated with the other clusters are mostly already silenced (Extended Data Fig. 4c). Genes associated with the low group are implicated in cell maintenance processes such as translation, replication and cell cycle, which explains why they continuously stay unmethylated. Genes with promoter CGIs of cluster 1 are enriched in MAPK and JNK signaling, two pathways frequently misregulated in cancer (Extended Data Fig. 4d). Comparing the chromatin state proportions of HSCs and DND41 for the variable CGI clusters 1 to 4, we observed that gain in CGI methylation was associated with an increase in the fraction of quiescence and heterochromatin in the cancer state. In contrast, bivalent regions were almost entirely lost in DND41 for clusters 2 to 4, which resembles previous findings on chromatin signature changes during tumorigenesis^43^. The changes between HSC and DND41 chromatin states were statistically significant for all CGI groups; however, only cluster 1 to 3 exhibited a high effect size, which suggests that the strongest change in chromatin state proportions between healthy and cancerous cells affects these clusters (chi-squared *P* values <1.7 × 10^−10^ in Supplementary Table 9). Of note, the overall trend from low to high methylation across the four clusters is consistent across other hematopoietic and solid tumors, suggesting a potential pan-cancer mechanism that leads to specific hypermethylation levels of shared CGIs (Fig. 4f and Extended Data Fig. 4e).

### The relationship of T-ALL CGI methylation with covariates

**Fig. 5:**
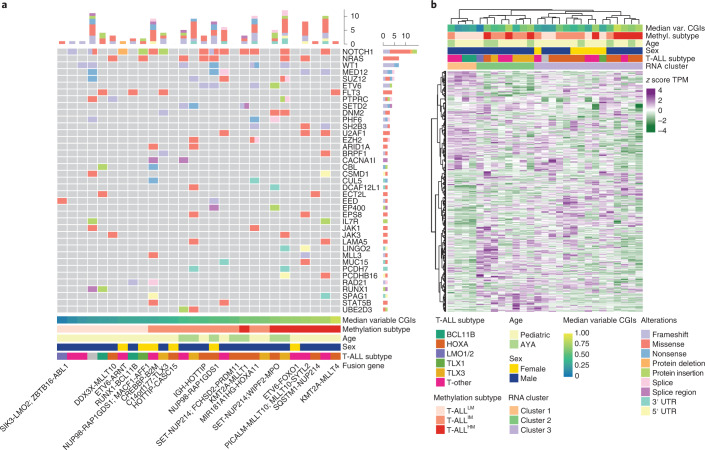
Characterization of possible drivers in patients with T-ALL. **a**, Oncoprint showing genes with mutations in at least two T-ALL samples ordered by frequency. Mutation types are stratified. Bottom row indicates fusion genes per sample, if present. **b**, Hierarchical clustering using Ward’s distance based on the 500 most variable expressed genes across patients with T-ALL (*n* = 27 patients). Samples mainly group by genetic/transcriptomic subtype (such as TLX3). The methylation-based subtypes cannot be fully recapitulated, although RNA-based cluster 3 shows a tendency for higher methylated samples (not significant, *P* = 0.38, two-sided Fisher’s exact test).

**Extended Data Fig. 5:**
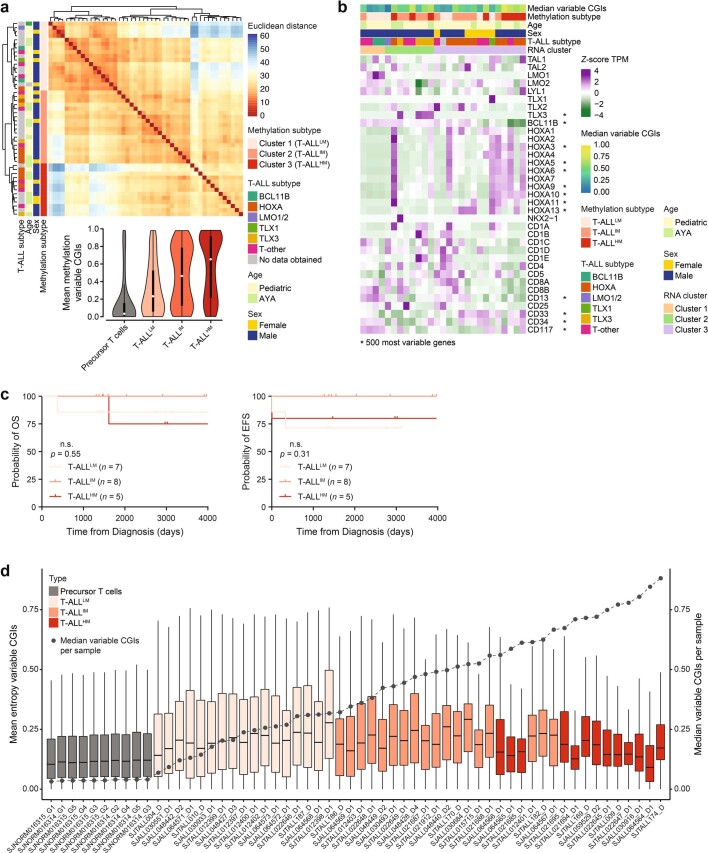
Methylation-based subtyping of T-ALL patients. **a)** Hierarchical clustering with Euclidean distance of T-ALL patients based on commonly covered variable CGIs (*n* = 8,863, top). Violin plots (bottom) show the distribution of variable CGIs in the three main clusters compared to healthy precursor T cells. White dots denote the median, edges denote the IQR and whiskers denote either 1.5 × IQR or minima/maxima (if no point exceeded 1.5 × IQR; minima/maxima are indicated by the violin plot range). **b)** Heatmap of standardized, log2-transformed expression for T-ALL patients and a selection of T-ALL marker genes. **c)** Event-free and overall survival for T-ALL patients of different methylation-based groups (information not available for all samples with WGBS data). No significant difference in outcome was detected (log-rank test). **d)** Boxplots of DNA methylation entropy across commonly covered variable CGIs (*n* = 8,863) for every healthy T-cell and T-ALL patient sample. Lines denote the median, edges denote the IQR and whiskers denote either 1.5 × IQR or minima/maxima (if no point exceeded 1.5 × IQR; outliers were omitted). The entropy is highest for samples of T-ALL^LM^ and T-ALL^IM^. The line indicates the median variable CGI methylation per sample (right y-axis).

We clustered all T-ALL samples based on their CGI methylation levels (hierarchical clustering using Euclidean distance between *n* = 8,863 commonly covered, variable CGIs across precursor T cell and T-ALL samples; Extended Data Fig. 5a). We then used the three main clusters representing the most extreme (low and high) and intermediate CGI methylation levels, termed T-ALL^LM^ (cluster 1), T-ALL^IM^ (cluster 2) and T-ALL^HM^ (cluster 3) to test the association of covariates with T-ALL samples presenting with different CGI methylation levels (Extended Data Fig. 5a). We did not detect any significant associations between T-ALL methylation-based groups and genetic subtypes, age, sex or recurrent mutations (Fisher’s exact test, *P* values >0.05 in Supplementary Tables 10 and 11 and Fig. 5a), although samples with the HOXA or TLX3 subtype seemed to more frequently co-occur with T-ALL^IM^ and T-ALL^HM^ groups in line with previous findings^31^. Clustering of patients with T-ALL based on the 500 most variably expressed genes, including previously described T-ALL marker genes, did not align with the methylation-based clustering of patients with T-ALL (Fig. 5b, Extended Data Fig. 5b and Supplementary Table 12). Instead, transcriptome-based clusters separate samples according to their genetic subtype. In contrast to previous studies, these methylation groups were not associated with clinical outcome, but our cohort was not of sufficient size to permit a rigorous multivariable analysis of clinical features, genomic alterations, methylation state and outcome (Extended Data Fig. 5c). However, T-ALL^LM^ and T-ALL^IM^ samples exhibited significantly higher intratumor methylation heterogeneity measured using DNA methylation entropy (high entropy reflects high heterogeneity), which may be relevant as higher entropy has been associated with poor prognosis in other cancers, including B-cell lymphoma^44,45^ (Extended Data Fig. 5d, *P* = 2.3 × 10^-6^ and *P* = 0.0002, respectively, Wilcoxon rank-sum test).

### Epigenetic regulators impact methylation levels in T-ALL

**Fig. 6:**
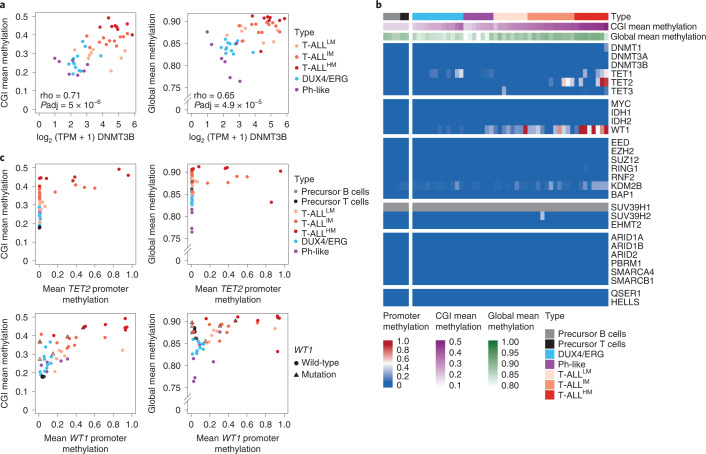
Impact of epigenetic regulators on global and CGI methylation in ALL. **a**, Correlation of CGI and global mean methylation with DNMT3B expression across patients with T-ALL, Ph-like and *DUX4*/*ERG* (*n* = 27, 7 and 12 patients, respectively). Correlation was calculated using a two-sided Spearman correlation test (rho, Spearman correlation) and *P* values were adjusted for multiple testing correction using false discovery rate (FDR) (*P*adj, adjusted *P* value). **b**, Heatmap showing the methylation status of the promoter CGIs of a panel of epigenetic regulators associated directly and indirectly with DNA methylation. Only *ARID1B* does not have a promoter CGI and instead the mean methylation of the promoter region is shown (1.5 kb upstream and 500 bp downstream of the TSS). *SUV39H1* is located on the X chromosome, which is excluded from methylation analysis due to the difference in methylation between female and male samples (inactivated allele is fully methylated in females). **c**, Correlation of *TET2* (top) and *WT1* (bottom) promoter methylation with mean CGI (left) and global methylation (right) levels across patients with T-ALL, Ph-like and *DUX4*/*ERG* with precursor B and T cells as control (*n* = 27, 7, 12, 4 and 2 samples, respectively). A subset of patients with T-ALL^IM^ and T-ALL^HM^ shows hypermethylation of the *TET2* and/or *WT1* promoter accompanied by increased average CGI and global methylation levels. *WT1* promoter hypermethylation is largely mutually exclusive with *WT1* mutations.

**Extended Data Fig. 6:**
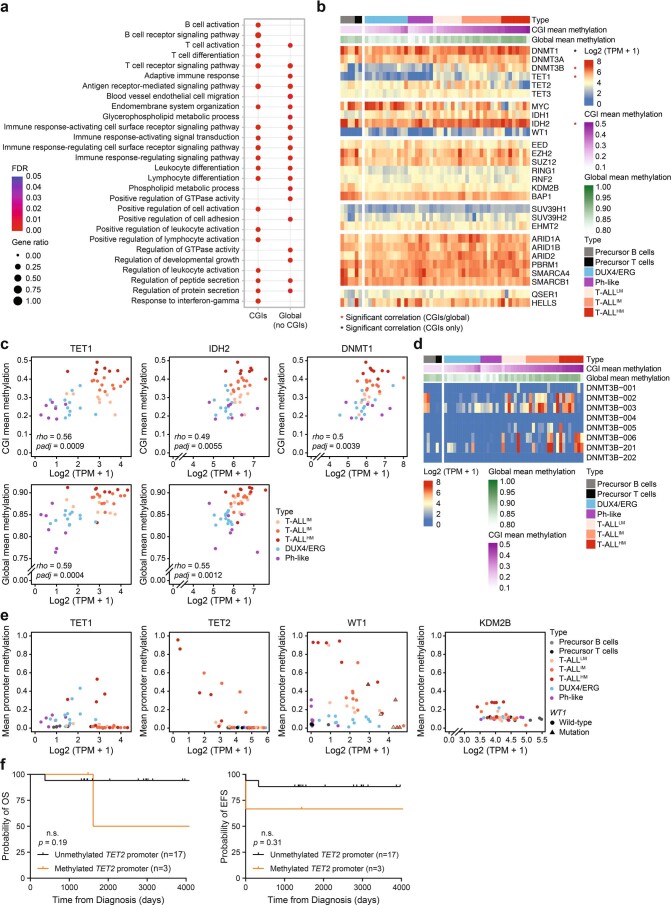
Relation of promoter hypermethylation and expression of epigenetic regulators in ALL. **a)** Overrepresentation analysis of genes significantly correlating with mean CGI and/or global methylation across T-ALL, DUX4/ERG and Ph-like patient samples (*n* = 1,833 and 1,898 genes respectively, *n* patients as in Fig. 6a). **b)** Heatmap showing the log2-transformed expression values per sample for a set of epigenetic regulators directly or indirectly associated with DNA methylation. **c)** Correlation of CGI mean methylation (top) with TET1, IDH2 and DNMT1 expression across T-ALL, Ph-like and DUX4/ERG patients as well as correlation of global mean methylation (bottom) with TET1 and IDH2 (*n* patients as in Fig. 6a). Correlation was obtained using a two-sided spearman correlation test (*rho* = spearman correlation) and *p*-values were adjusted for multiple testing correction using FDR (*padj* = adjusted *p*-value). **d)** Heatmap showing the log2-transformed expression of different DNMT3B isoforms. The catalytically active isoforms DNMT3B-002 or to a lower extent DNMT3B-001 are expressed in most T-ALL patients. **e)** Correlation of TET1, TET2, WT1 and KDM2B expression with the mean methylation of the respective promoter CGIs (*n* patients as in Fig. 6c). *TET2* and *WT1* promoter hypermethylation are associated with decreased gene expression while *TET1* and *KDM2B* hypermethylation does not seem to affect the expression level consistently. **f)** Event-free and overall survival for T-ALL patients with and without *TET2* promoter hypermethylation (information not available for all samples with WGBS data). No significant difference in outcome was detected (log-rank test).

Despite the variability and generally higher level in global as well as CGI methylation levels across patients with T-ALL compared to B-ALL samples, we did not detect recurrent mutations in epigenetic regulators (Fig. 5a). We therefore next conducted a correlation test of both global and CGI average CpG methylation with the expression of each gene across T-ALL, *DUX4*/*ERG* and Ph-like B-ALL samples with available expression data (using Spearman correlation, selected candidates with adjusted *P* value <0.01; Supplementary Tables 13 and 14). Hypodiploid samples were omitted due to confounding effects of the high aneuploidy. Overall, 1,898 genes were significantly correlated for our global comparison and 1,833 for our CGI-based comparison (1,390 genes are shared between both). Due to the higher methylation levels in T-ALL, many significantly correlated genes were enriched in B or T lymphocyte-specific pathways (Extended Data Fig. 6a). However, when examining epigenetic regulators associated with DNA methylation, we found that expression of DNMT3B was significantly positively correlated with global as well as average CGI methylation levels (Fig. 6a and Extended Data Fig. 6b,c). Expression of DNMT3B includes expression of the catalytically active isoforms DNMT3B-002 and, to a lesser extent, DNMT3B-001, in T-ALL samples, which are usually inactive in adult somatic cells^46^ (Extended Data Fig. 6d). We did not observe a significant correlation of previously reported genes with CGI or global levels such as MYC. IDH2 correlates with both CGI and global methylation levels; however, so far it has been described to cause hypermethylation due to mutation, not expression differences^12^. To identify additional candidates that contribute to the high methylation landscape in T-ALL, which may have been missed if they occur only in a small subset of patients, we examined the promoter status of a panel of epigenetic regulators (Fig. 6b). We observed hypermethylation (promoter CGI methylation >0.2) of the *TET2* promoter in 26% of patients with T-ALL, resulting in a decrease or complete loss of TET2 expression and coinciding with high overall CGI methylation (Fig. 6b and Extended Data Fig. 6b,c). Additionally, we observed rare hypermethylation of the *TET1* promoter, highly coinciding with *TET2* promoter hypermethylation. Notably, promoter hypermethylation associated with reduced expression was also visible for *WT1*, a tumor suppressor in T-ALL and generally occurred in the absence of *WT1* mutations. This was observed in patients with high global and CGI methylation levels and frequently with *TET2* hypermethylation (Fig. 6b,c and Extended Data Fig. 6e). WT1 recruits TET2 in AML, which could indicate a coupled mechanism behind the loss of both genes in T-ALL^47^. As before due to sample size, we could not detect significantly different outcomes between patients with and without *TET2* promoter hypermethylation (Extended Data Fig. 6f).

### TET2 disruption influences methylation in T-ALL cell lines

**Fig. 7:**
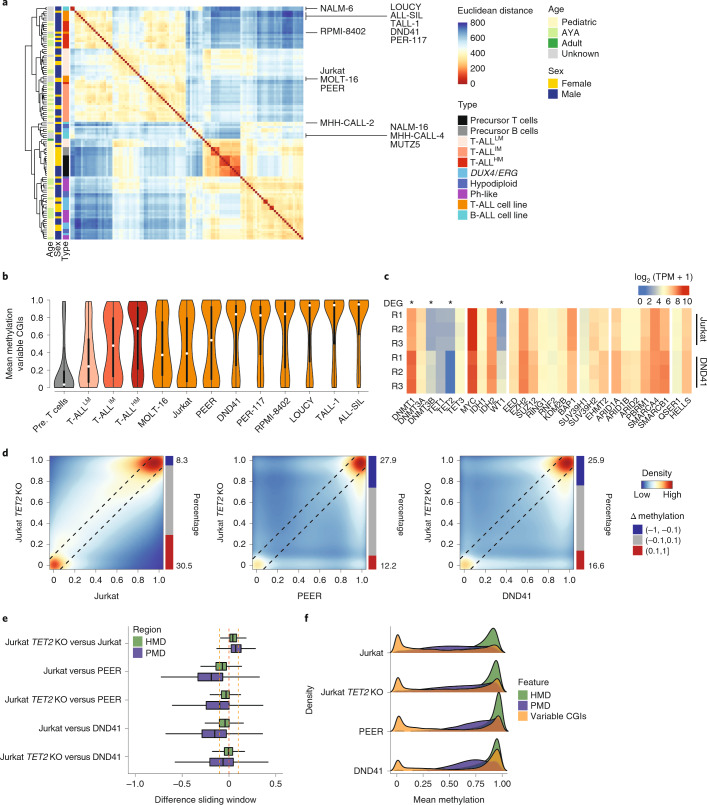
TET2 deletion in Jurkat cells influences hypermethylation. **a**, Hierarchical clustering with Euclidean distance of all patients with ALL, healthy control and cell line samples (*n* samples as shown in Fig. 1a) based on the 5% most variable CpGs covered by at least 80% of samples (*n* = 999,470 CpGs). Samples largely separate by lineage and cell lines overall group to primary samples of the same origin. **b**, Violin plots showing the mean methylation of variable CGIs (*n* = 9,349 CGIs, defined across all healthy and ALL samples) for each T-ALL cell line and the T-ALL methylation-based subtypes as well as healthy cells for comparison. White dots denote the median, edges denote the IQR and whiskers denote either 1.5 × IQR or minima/maxima (if no point exceeded 1.5 × IQR; minima/maxima are indicated by the violin plot range). **c**, Heatmap of epigenetic regulators and their expression status (log_2_-transformed TPM) in Jurkat and DND41. TET2 is significantly upregulated in Jurkat (adjusted *P* = 6.35 × 10^−27^), whereas DNMT1, DNMT3B and WT1 are significantly downregulated in comparison to DND41 (adjusted *P* = 2.79 × 10^−48^, 3.46 × 10^−44^ and 7.41 × 10^−88^, respectively; two-sided Wald test). **d**, Pair-wise correlation of CpG methylation levels of Jurkat, Jurkat *TET2* KO, PEER and DND41 (blue, low density; red, high density). Black lines mark the difference of 0.1 from the diagonal in both directions. No replicates were generated. **e**, Box plot of methylation differences across commonly covered sliding windows comparing Jurkat WT and *TET2* KO split by HMD and PMD (*n* = 3,658,861 and 4,855,565 windows, respectively; properties are as in Fig. 2a, covered by all samples considered). Lines denote the median, edges denote the IQR and whiskers denote either 1.5 × IQR or minima/maxima (if no point exceeded 1.5 × IQR; outliers were omitted). *TET2* deletion leads to a gain of methylation in both HMDs and PMDs, which is reaching similar levels compared to DND41 in HMDs, whereas PMDs still remain lower. **f**, Distribution of methylation of 1-kb tiles in HMDs and PMDs (*n* = 919,527 and 1,216,886 tiles, respectively) as well as variable CGIs (*n* = 8,863 CGIs) for Jurkat samples with and without *TET2* KO as well as compared to DND41 and PEER as control, highly methylated cell lines.

**Extended Data Fig. 7:**
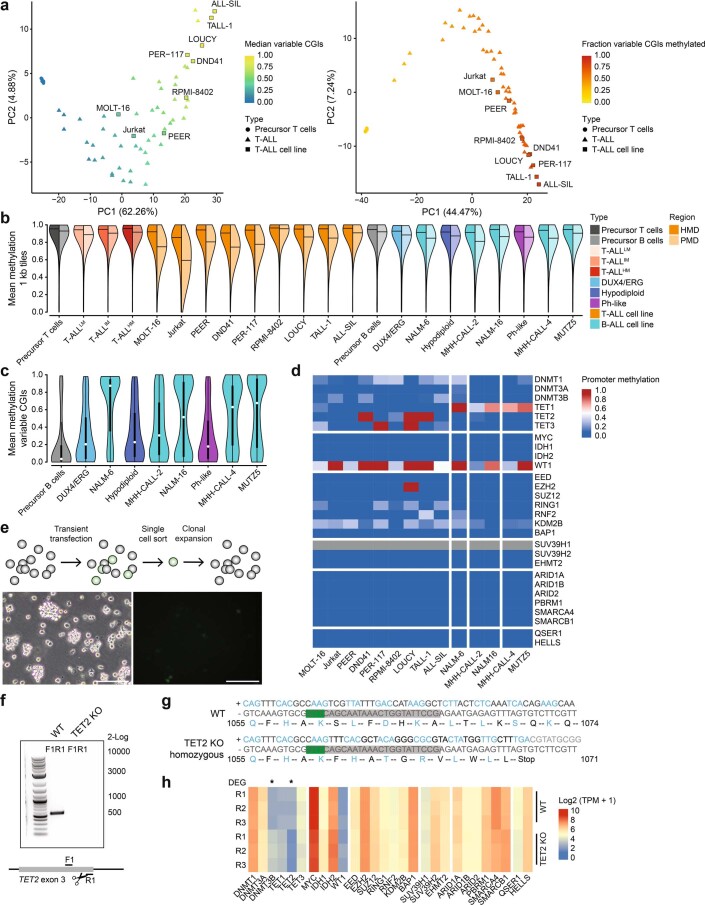
Comparison of ALL patients and cell lines. **a)** PCA based on the average methylation of the variable commonly covered CGIs (left) and based on the methylation status of the variable commonly covered CGIs (right) of precursor T cells, T-ALL patients and T-ALL cell lines (*n* = 7,914 CGIs, *n* = 10 precursor T cell, 48 T-ALL and 9 cell line samples). **b)** Violin plot showing the mean methylation of 1-kb tiles separated by HMDs and PMDs (*n* = 718,674 and *n* = 716,821 tiles respectively) for each T-ALL and B-ALL cell line as well as ALL subtypes and healthy cells for comparison. Lines denote the median. **c)** Violin plot showing the mean methylation of variable CGIs (defined across all normal and ALL samples, *n* = 9,349) for each B-ALL cell line and B-ALL subtypes as well as healthy cells for comparison. White dots denote the median, edges denote the IQR and whiskers denote either 1.5 × IQR or minima/maxima (if no point exceeded 1.5 × IQR; minima/maxima are indicated by the violin plot range). **d)** Heatmap showing the methylation status of the promoter CGIs of a panel of epigenetic regulators in T-ALL and B-ALL cell lines. Only *ARID1B* does not have a promoter CGI and instead the mean methylation of the promoter region is shown. *SUV39H1* is located on the X chromosome, which is excluded from methylation analysis. **e)** Jurkat cells were transfected with px458 containing a guide RNA targeting exon 3 of the *TET2* gene and expressing a GFP reporter. GFP positive cells were sorted by FACS as single cells and expanded. Images show transfected Jurkat cells in brightfield (left) and the GFP signal (right). The transfection was repeated four times independently leading to one successful knockout. The image is representative of four images taken from one plate. Scale bar = 100 um. **f)** cDNA genotyping PCR in WT and Jurkat *TET2* KO cells. A primer pair (F1, R1) inside exon 3 and flanking the target site of the gRNA yields a 525 bp product in WT Jurkat cells, and no product in the KO when run on a 1% agarose gel with the NEB 2-Log DNA ladder. **g)** Jurkat *TET2* KO shows a 7-kb insertion at the guide RNA cut site in exon 3, which is normally 3,455 bp long in WT cells, resulting in a premature stop codon. **h)** Expression of epigenetic regulators in Jurkat WT and KO cells. Upon *TET2* KO, DNMT3B gets significantly up-regulated. Source data

To examine the contribution of TET2 in shaping the methylation landscape, we first sequenced a panel of T- and B-ALL cell lines to assess fidelity to the parental leukemia subtypes. Three T-ALL cell lines (MOLT-16, Jurkat and PEER) recapitulate intermediate CGI methylation levels of patients with T-ALL^IM^, whereas six other cell lines rather reflect patients with T-ALL^HM^ (DND41, PER-117, RMPI-8402, LOUCY, TALL-1 and ALL-SIL) (Fig. 7a,b, Extended Data Fig. 7a). Globally, cell lines show lower methylation levels than primary tumors, which is most pronounced for Jurkat (Extended Data Fig. 7b). For B-ALL, only MHH-CALL-2 presents with CGI methylation levels similar to the respective B-ALL subtypes (Extended Data Fig. 7c). All other cell lines exhibit elevated CGI methylation.

High CGI methylation in the six T-ALL cell lines could reflect similar epigenetic regulation as T-ALL^HM^; however, it could also be caused by culturing effects as reported previously^48,49^. We therefore examined the promoter methylation status of *TET2*, *WT1* and other epigenetic regulators in all cell lines. DND41, TALL-1 and LOUCY show hypermethylation of both promoters similar to some patients with T-ALL^HM^ (Extended Data Fig. 7d). As a control, B-ALL cell lines show an unmethylated *TET2* promoter in line with primary patient data. We profiled one T-ALL^IM^-like and one T-ALL^HM^-like cell line (namely Jurkat and DND41) using RNA-seq and found that TET2 is expressed in Jurkat, but not DND41, which is consistent with the promoter methylation (Fig. 7c and Supplementary Table 15). Additionally, DNMT3B is significantly upregulated in DND41. To characterize the contribution of TET2 further, we knocked out *TET2* in Jurkat cells and performed WGBS and RNA-seq (Extended Data Fig. 7e–g). Comparing Jurkat knockout (KO) with wild-type (WT) cells, a gain of methylation specifically at already highly methylated CpGs is visible (Fig. 7d). More detailed comparisons of the genome-wide methylation change based on sliding windows (excluding CGIs) show a global background methylation gain compared to WT cells, where HMDs reach levels of DND41; however, PMDs remain still less methylated compared to PEER or DND41 (Fig. 7e). The level of average CGI methylation rose compared to WT cells to levels similar to PEER but so far remained lower than the extreme CGI methylation levels of DND41 (Fig. 7f). This suggests a partial contribution of TET2 in Jurkat cells on CGIs (average CpG methylation 0.25 and 0.43 for WT and KO, respectively with 1.2–1.7 million affected CpGs) as well as on the global methylation landscape (average CpG methylation 0.67 and 0.74 for WT and KO respectively with 22–24 million affected CpGs). Differential expression between Jurkat *TET2* KO and WT cells revealed upregulation of DNMT3B, which correlates with global and CGI methylation levels across ALL subtypes (Extended Data Fig. 7h and Supplementary Table 16).

## Discussion

Our comprehensive, high-coverage genome-wide methylation dataset of patients with ALL, healthy control samples and ALL cell lines demonstrates important features and insights into the distinguishing methylation landscape of ALL compared to most other hematopoietic as well as solid malignancies. First, we show that the global DNA methylation landscape of ALL and more specifically patients with T-ALL deviates from the canonical cancer methylome and only exhibits local hypermethylation without the typical global loss of methylation. The only other tumor type for which we could observe a similarly highly methylated genome is AML. In contrast to chronic leukemias, both acute leukemia types are characterized by the accumulation of immature cells of different blood lineages (lymphoid versus myeloid). As the cells of origin for both AML and ALL are premature in contrast to other hematopoietic malignancies such as mutated CLL and MCL, this could indicate a connection between cell of origin stage and global methylation loss. However, TPLL exhibits global hypomethylation despite the mature T cells of origin showing a highly methylated genome comparable to that of thymic precursor T cells. We also note a difference between B- and T-ALL, which should arise from similar immature stages, that we cannot explain yet but that may hold relevant insights.

Global hypomethylation has been hypothesized to occur progressively in late-replicating domains and might therefore be prominent in cancer due to an increase in the number of mitotic cell divisions^9^, although why this does not affect the highly proliferative ALLs and AMLs is not clear. A more recent study in colorectal cancer suggested that global hypomethylation and associated topological changes occur as a defense mechanism of the cell rather than a tumor-promoting mechanism^10^. In both ALL and AML, the disease tends to progress rapidly, often only within weeks. The lack of hypomethylation might be associated with the failure of the cells to initiate this tumor-suppressive response or the ability of emerging tumor cells to deactivate that mechanism. Therefore, in the context of this recently proposed model, the absence of global hypomethylation in these two tumor types provides a path toward better understanding of the underlying regulation.

We further show that the CGI hypermethylation range of patients with T-ALL follows a dynamic pattern instead of a clear separation into previously defined CIMP groups. CIMP has been traditionally characterized based on the DNA methylation of a panel of marker genes or variable CpG probes on the Illumina Infinium Array (usually around 1,000 CpGs)^50–52^. These approaches do not consider the methylation levels across CGIs in general and therefore provide only limited interpretability. Although CIMP has been defined in various cancer types such as breast cancer^51^, colon cancer^53^, ALL^54^ and glioma^52^, the overall levels of CGI methylation in each CIMP-positive or -negative class remain elusive as the classification is based only on a small fraction of CpGs in a limited set of CGIs defined explicitly for each tumor type.

On the basis of the continuous CGI methylation levels across our patients with T-ALL, we were able to identify CGIs that seem to be frequent targets of hypermethylation across all samples. Notably, the vast majority of these preferentially hypermethylated CGIs did not affect active promoters recapitulating previous findings on cancer hypermethylation^55,56^ and aberrant methylation affecting active promoters seemed to follow a rather sample-specific pattern. Our analysis gives insights into groups of CGIs that are systematically targeted to different extents of hypermethylation, which is reflected in a pan-cancer comparison. We previously reported CGIs that are hypermethylated during development in the extraembryonic lineage and commonly methylated across a broad range of cancer types^57^. Together these findings could indicate that although CGI hypermethylation has been partially observed to be tumor- or subtype-specific, a pan-cancer mechanism exists that organizes CGIs into preferential targets, which can then acquire distinct levels of methylation.

Previous studies have not been able to link the variable CGI hypermethylation in T-ALL to a recurrently mutated or dysregulated epigenetic regulator. A recent study associated patients with T-ALL with increased levels of CGI hypermethylation to the aging of thymocytes during a preleukemic phase and therefore, to the age of the cells of origin based on comparisons between patients and mouse models of T-ALL^32^. Comparing pediatric and adult patients should mimic some aspects of an age-related epigenetic mechanism; however, we could not observe an enrichment of adolescent or adult patients in T-ALL^IM^ and T-ALL^HM^ in comparison to T-ALL^LM^. We also could not link T-ALL CGI methylation levels to common mutations in line with previous findings. As we found a more widespread CGI hypermethylation trend, a direct comparison between groups defined for simplicity might not uncover potentially associated, dysregulated genes; however, we observed *TET2* promoter hypermethylation in a subset of patients with T-ALL^IM^ and T-ALL^HM^, leading to decreased TET2 expression. The possible involvement of TETs suggests that at least part of the observed CGI hypermethylation may represent hydroxymethylation, which is not distinguishable from methylcytosine by WGBS. Additionally, counteracting expression of DNMT3s and TETs might increase methylation turnover at CGIs^58^. Upon loss of TET2 in some patients, the dynamics could be biased toward de novo methylation leading to increased DNA methylation levels in general.

Finally, although our selection of T-ALL cell lines seemed to resemble parts of the DNA methylation dynamics in patients with T-ALL, their respective methylome still deviates from primary T-ALL cases, most notably by lower genome-wide methylation levels likely reflecting culture-induced changes. Together these findings highlight that some cancer cell lines can serve as a model for certain aspects of in vivo methylation dynamics; however, they should be used with care as the DNA methylation landscape and epigenetic machinery is frequently compromised.

Our study provides important data and insights into the non-canonical epigenetic regulation of ALL as well as cancer more generally that, combined with recent studies, helps to focus our attention on particular aspects of the cancer methylome and will help us move toward an improved mechanistic understanding of the common epigenetic changes in cancer.

## Methods

### Patients and samples

Diagnosis leukemia samples were obtained from children treated on St. Jude Children’s Research Hospital and Eastern Cooperative Oncology Group protocols. Patients and/or guardians provided informed consent/assent and the study was approved by the Institutional Review Board of St. Jude Children’s Research Hospital. Patients were not compensated. The leukemia samples were obtained by bone-marrow aspiration at diagnosis. All samples were purified by density gradient centrifugation using Ficoll and were of at least 90% tumor purity before DNA/RNA extraction. Healthy T cells were flow-sorted from neonatal thymi collected at the time of cardiac surgery. B cells were flow-sorted from healthy childhood bone marrow collected at bone-marrow donation for allogeneic transplantation into fractions based on CD34, CD19 and sIg into pro, pre and mature B cells. Control cells were not paired to presented leukemia samples. Patients included both males and females and were grouped by age into pediatric patients (0–15 years), AYAs (16–39 years) and adults (≥40 years). Exact details of demographic data of each participant are included in Supplementary Table 1.

### Genomic sequencing

Whole-genome, transcriptome and exome sequencing was performed using Illumina exome baits and library preparation and Illumina Hi-Seq or NovaSeq sequencers as previously described^59,60^. For transcriptome sequencing of T-ALL cell lines, RNA was extracted from cells using the QIAGEN RNeasy Mini kit (74104) and the quality was assessed on the Agilent TapeStation. RNA-seq libraries were then prepared using the KAPA Stranded mRNA-seq kit (KK8420, Roche, 07962193001). The resulting libraries were cleaned using the Agencourt AMPure XP system (Beckman Coulter, A63881) to remove all adaptor dimers and the quality of the final libraries was then assessed for using the Agilent TapeStation. The libraries were sequenced on the NovaSeq6000, generating 100-bp paired-end reads.

WGBS of primary patient samples and all cell lines except Jurkat and DND41 was performed on a bisulfite-modified DNA-sequencing library generated according to Illumina’s instructions accompanying the TruSeq DNA Methylation kit (part EGMK81312). A total of 200 ng of genomic DNA, including 0.2% Lambda DNA (N6-methyladenine-free; NEB) was bisulfite-converted in a single reaction using the EZ DNA Methylation Gold kit (Zymo Research) as outlined by the manufacturer. The bisulfite-converted DNA (bsDNA) was split into four aliquots per sample. Four single indexed WGBS libraries were constructed with the EpiGnome Methyl-Seq kit (EGMK81312, Epicentre) according to the manufacturer’s recommendations. The final concentration of each library was accurately determined through qPCR (KAPA Biosystems). The four independent libraries were normalized, pooled and loaded across three lanes of the Hi-Seq 2000 instrument (Illumina). For each library pool, 2 × 101-bp paired-end sequence data were generated.

For Jurkat (WT and *TET2* KO) and DND41, genomic DNA was extracted using the PureLink Genomic DNA Mini kit (Thermo Fisher, K182002) following manufacturer’s instructions. gDNA was then sheared in Covaris micro TUBE AFA Fiber Pre-Slit Snap-Cap tubes (SKU, 520045) and purified using the Zymo DNA Clean & Concentrator-5 kit (D4013) according to manufacturer’s instructions. Sheared gDNA was then bisulfite-converted using the EZ DNA Methylation Gold kit (Zymo, D5005) and processed using the Accel-NGS Methyl-seq DNA library kit (Swift Biosciences, DL-ILMMS-12) both following manufacturer’s recommendations. Libraries were prepared and cleaned using Agencourt AMPure XP system (Beckman Coulter, A63881) and the absence of adaptors was verified using the Agilent TapeStation. The final libraries were sequenced on the NovaSeq6000 yielding 150-bp paired-end reads.

### Cell culture

Jurkat (DSMZ, ACC 282) and DND41 (DSMZ, ACC 525) cells were cultured in RPMI 1640 GlutaMAX supplement (Thermo Fisher, 61870044) with 10% FBS. Cells were maintained in suspension culture at a density between 0.3 × 10^6^ and 1 × 10^6^. Cells were split at a ratio of 1:2 to 1:4 every 2–4 d and were frozen in 70% RPMI medium, 20% FBS and 10% dimethylsulfoxide. PEER (ACC6, DSMZ), PER-117 (a gift from U. Kees, Perth), MOLT-16 (ACC29, DSMZ), RPMI-8402 (ACC290, DSMZ), LOUCY (ACC394, DSMZ), TALL-1 (ACC521, DSMZ), ALL-SIL (ACC511, DSMZ), NALM-6 (ACC128, DSMZ), NALM-16 (ACC680, DSMZ), MHH-CALL-2 (ACC341, DSMZ), MHH-CALL-4 (ACC337, DSMZ) and MUTZ5 (ACC490, DSMZ) were maintained in RPMI 1640 medium containing 10% or 20% FBS (HyClone), penicillin/streptomycin (100 U ml^−1^) and glutamine (100 µM) at 37 °C, 5% CO_2_. Cell identity was confirmed by short tandem repeat profiling using a PowerPlex Fusion System (Promega). All cell lines were confirmed as free from *Mycoplasma* spp. using the Universal Mycoplasma Detection kit (American Type Culture Collection).

### Generation of TET2 KO Jurkat cells

Jurkat cells were transfected with px458 (Addgene plasmid no. 48138) containing a guide RNA (target sequence: CTTATGGTCAAATAACGACT) targeting exon 3 of the *TET2* gene, near the beginning of the catalytic domain^61^ and expressing a GFP reporter. The transfection was carried out using the Amaxa 4D nucleofector X-Unit (Lonza) following manufacturer’s recommendations. GFP-positive cells were sorted by FACS as single cells into a 96-well plate for clonal expansion and screening. Percentages of sorted cells were analyzed using FlowJo (v.10.3). Disruption of the targeted locus was verified by genotyping PCR and Sanger sequencing (primer pair: forward GTCTGGTCAACAAGCTGCGC, reverse AAAGCTGGGGTGTGGCTATC).

### Whole-genome bisulfite sequencing processing

Paired-end reads from sequencing were trimmed using trimgalore (v.0.4.4, https://www.bioinformatics.babraham.ac.uk/projects/trim_galore/), removing low-quality (Q30) bases and adaptor sequences and clipping ten bases from each end of the read pairs. Trimmed reads were then mapped to hg19 using BSMAP (v.2.90) with default parameters to report unique pairs with a 17-bp minimal insert size and 600-bp maximum insert size^62^. Duplicates were removed using the ‘MarkDuplicates’ command from GATK (v.4.1.4.1;--VALIDATION_STRINGENCY = LENIENT--REMOVE_DUPLICATES = true)^63^. DNA methylation rates were called using mcall from the MOABS package (v.1.3.2; --excludedFlag 512)^64^. All analyses were restricted to autosomes and only CpGs with a minimum coverage of 10 and a maximum coverage of 150 reads were considered.

### RNA-seq processing

Raw reads were subjected to adaptor and quality trimming with cutadapt (v.2.4; parameters: --quality-cutoff 20 --overlap 5–minimum-length 25 --interleaved --adaptor AGATCGGAAGAGC -A AGATCGGAAGAGC), followed by poly-A trimming with cutadapt (parameters: --interleaved --overlap 20 --minimum-length–adaptor ‘A[100]’ --adaptor ‘T[100]’). Reads were aligned to the human reference genome (hg19) using STAR (v.2.7.5a; parameters: --runMode alignReads --chimSegmentMin 20 --outSAMstrandField intronMotif --quantMode GeneCounts)^65^ and transcript expression was quantified using stringtie^66^ (v.2.0.6; parameters: -e) with GENCODE annotation (release 19). Promoters were defined as 1,500 bp upstream and 500 bp downstream of the annotated TSS. The annotated gene coordinates were defined as gene bodies.

### Fusion gene calling and ALL subtype definition

Chromosomal rearrangements, fusions and ALL subtypes were defined as previously described^60,67^.

### External data

Methylation rates and coverage information for WGBS data of eight different solid tumor types and corresponding healthy tissue from TCGA was downloaded from https://zwdzwd.github.io/pmd. Methylation rates and coverage information for WGBS data of blood malignancies and healthy blood samples was downloaded from the Blueprint epigenome project and coordinates of CpGs were lifted to hg19. Only lifted positions matching a CpG in the hg19 reference genome were considered for further analysis. All pre-processed methylation rates were filtered for CpGs covered by at least 10 and at most 150 reads. Sample IDs and sources are listed in Supplementary Table 2. Annotation of PMDs and HMDs as well as solo-WCGW CpGs was downloaded from https://zwdzwd.github.io/pmd.

### Bioinformatic analysis

All analyses were carried out using R 3.6.3.

#### Global DNA methylation analyses

The annotation of CGIs for hg19 was downloaded from UCSC. CGI shores were defined as the 2 kb flanking a CGI on each side, whereas shelves were defined as the 2 kb flanking the shores. Tiles of 1 kb size were obtained by segmenting the genome hg19 with bedtools ‘makewindows’^68^. Methylation for each CGI or tile per patient was calculated as the arithmetic mean of CpGs overlapping the CGI/tile. Overlap of CpGs with features was calculated using bedtools ‘intersectBed’. Only features covered by at least three CpGs were considered for further analyses.

The global level of DNA methylation per sample was determined using the arithmetic mean of the methylation rates of all CpGs outside of CGIs covered per sample (autosomes only). The average CGI methylation level per sample was determined using the arithmetic mean of the methylation rates of all CpGs inside of CGIs covered per sample.

WGBS control, patient and cell line samples were hierarchically clustered based on the 5% most variable CpGs covered by at least 80% (999,470 CpG) of samples with Euclidean distance using the pheatmap package^69^.

#### Subtype-specific DNA methylation

Average methylation per subtype (ALL, solid tumor, other hematopoietic malignancy or control subtypes) was calculated by computing the average CpG methylation level for every CpG covered by at least 80% of the samples of the respective subtype. These per-CpG averages were then used to calculate averages in sliding windows (excluding CpGs in CGIs, Fig. 2a), 1-kb tiles (Extended Data Fig. 7b) and CGIs (Fig. 7b and Extended Data Fig. 7c).

#### Sliding window analysis

Sliding windows of the reference genome were computed using bedtools ‘makewindows’ with options ‘-w 1,000 -s 250’ only considering autosomes. Methylation for each sliding window per patient was calculated as the arithmetic mean of CpGs overlapping the window, excluding CpGs in CGIs. Only sliding windows covered by at least three CpGs were considered for further analyses. Hypomethylated and hypermethylated sliding windows were defined as all windows showing a difference less than −0.1 and greater than 0.1, respectively when compared to the matching control. As controls, precursor B and T cell data generated within this study were used for B-ALL and T-ALL subtypes; memory B cells were used for CLL and MCL; HPCs were used for AML; CD4 and CD8 single positive alpha beta T cells were used for TPLL; and the respective healthy tissues were used for the solid tumors. Sliding windows were classified into HMDs or PMDs based on the largest overlap with predefined HMD/PMD regions (https://zwdzwd.github.io/pmd).

#### DNA methylation valley definition

For the T-cell and B-cell-specific DMV definition, sliding windows of the genome were calculated using bedtools ‘makewindows’ with options ‘-w 5,000 -s 1,000’ only for autosomes. The average methylation of each window was calculated using the subtype average CpG levels of either precursor T or B cells, excluding CpGs in CGIs. Additionally, the mean methylation of CGIs was calculated separately. Windows and CGIs with a mean methylation less than 0.15 and a minimum number of ten CpGs per region were merged if overlapping and termed DMVs (excluding regions that consisted only of CGIs without flanking regions).

#### T-ALL sample classification

Hierarchical clustering based on the variably methylated, commonly covered CGIs was carried out using the R package pheatmap with Euclidean distance. The top-level three clusters and their mean methylation levels were used to determine T-ALL^LM^, T-ALL^IM^ and T-ALL^HM^ samples.

PCA was carried out based on the level or methylation status (methylated >0.2, yes/no) of variably methylated, commonly covered CGIs across patients with T-ALL and precursor T cell samples (Fig. 4), as well as including T-ALL cell lines (Extended Data Fig. 7). Here, the PCA was calculated based on healthy cells and patient samples followed by the projection of cell line samples onto the PCA.

#### PMD and HMD heatmap

For each PMD and HMD, the arithmetic mean across solo-WCGW CpGs was calculated. Only regions covered by all samples were displayed.

#### DMR calling

DMRs were called using metilene (v.0.2–8; -m 10 -d 0.2 -c 1 -f 1 -M 300 -v 0.7). Only DMRs with a *q* value <0.05 were considered for further analysis. The B-ALL subtypes Ph-like and *DUX4*/*ERG* were tested against precursor B-cell samples while T-ALL was tested against precursor T cells. DMRs were annotated to overlap specific features (chromatin states, CGIs CGI shores and shelves, DMVs and PMDs) if either 20% of the DMR or 20% of the feature were overlapping using bedtools ‘intersectBed’. The chromatin states as annotated by ChromHMM were grouped the following way: Active TSS (1_TssA, 2_TssAFlnk), Bivalent TSS (10_TssBiv, 11_BivFlnk), Transcript (3_TxFlnk, 4_Tx, 5_TxWk), Enhancer (6_EnhG, 7_Enh, 12_EnhBiv), Heterochromatin (8_ZNF/Rpts, 9_Het), Repressive (13_ReprPC, 14_ReprPCWk) and Quiescent (15_Quies).

To create a set of random background DMRs, for each comparison (subtype-specific tumor and healthy samples) the regions that could potentially include a DMR based on the covered CpGs were extracted (consecutive CpGs not further than 300 bp apart). To create a set of random background DMRs, random regions with similar characteristics as the called DMRs were sampled 1,000 times from the genome (same lengths as called DMRs, minimum ten CpGs not further than 300 bp apart). The overlap with genomic features was calculated the same way as for the observed DMRs. The enrichment of DMRs per feature class was calculated by dividing the fraction of DMRs overlapping a feature class by the fraction of background DMRs overlapping the same class.

#### Clustering of CGIs

For the T-ALL specific clustering of CGIs, commonly covered CGIs between T-ALL samples, precursor T cells and T-ALL cell lines were selected. CGIs with an average methylation of less than 0.2 or higher than 0.8 in all samples were excluded from the clustering. CGIs were clustered based on the samples using the R package ‘ConsensusClusterPlus’ with parameters maxK, 12; reps, 100; pItem, 0.8; pFeature, 1; clusterAlg, ‘pam’; and distance, ‘Euclidean’. Four clusters were determined to be optimal based on the consensus matrix and were sorted on the basis of their average methylation levels (termed cluster 1 to 4 with increasing methylation levels). The clusters were visualized using the R package ComplexHeatmap^70^.

CGIs were determined to overlap with a specific feature (DMV, PMD, promoter and gene body) if either 20% of the CGI or 20% of the feature were overlapping. CGIs not overlapping promoters or gene bodies were termed intergenic. Active promoters were defined based on the average expression of precursor T cells (average TPM > 1). For chromatin states, CGIs were assigned to the state with the highest overlap.

#### DNA methylation entropy analysis

For all patients with T-ALL, entropy per 4-mer of CpGs was calculated using RLM^71^. Mean entropy per CGI was calculated using the arithmetic mean. Only 4-mers covered by at least 10 and at most 150 reads were considered.

#### Selection of epigenetic regulators

For mutation, promoter hypermethylation and expression analyses, we defined a set of epigenetic regulators that have been reported to directly or indirectly regulate/influence DNA methylation to screen candidates that could be involved in the unique methylation landscape of ALL and in particular patients with T-ALL. These include:

1. Direct DNA methylation regulators (DNA methyltransferases and TET enzymes): DNMT1, DNMT3A, DNMT3B, TET1, TET2 and TET3
2. Genes encoding proteins involved in recruitment/regulation of DNA methylation regulators, particularly in leukemia:
  MYC: reported to orchestrate changes in expression of DNA methylation-related enzymes in T-ALL in mouse models and cancer cell lines^33^
  WT1: shown to recruit TET2, which can be disrupted in AML^47^
  IDH1/2: if mutated can cause hypermethylation of CGIs^12^
3. Essential Polycomb group proteins: EED, EZH2, SUZ12, RING1, RNF2, KDM2B and BAP1
4. H3K9 histone methyltransferases: SUV39H1, SUV39H2 and EHMT2 (cross-talk between H3K9me3 and DNMT1 to stabilize proper methylation maintenance)^72^
5. Chromatin remodeling: ARID1A, ARID1B, ARID2, PBRM1, SMARCA4 and SMARCB1
6. Others:
  QSER1: shown to shield DMVs from DNA methylation^73^
  HELLS: modulates DNA methylation at genomic repeats. Loss leads to widespread hypomethylation^74^

#### RNA-seq analysis

Most variable genes across T-ALL samples were defined by calculating the s.d. per gene using log_2_-transformed TPMs. The log_2_-transformed TPMs of the top 500 variable genes were visualized using ComplexHeatmap with hierarchical clustering and Ward’s distance.

Differential gene expression was calculated using DESeq2 (ref. ^75^). Genes with an absolute log_2_ fold change >1 and an adjusted *P* value <0.05 were termed differentially expressed. Lowly expressed genes (average TPM < 0.5 across all considered samples) were excluded from the analysis.

Correlation between per-sample CGI and non-CGI mean methylation with gene expression (log_2_ TPM) across patients was carried out with the function ‘cor.test’ in R using the Spearman’s correlation. Lowly expressed genes (average TPM < 0.5 across all considered samples) were again excluded from the analysis. *P* values were corrected for multiple testing using FDR and genes with significant correlation were selected (adjusted *P* value <0.01).

#### Promoter DNA methylation analysis

The promoters of epigenetic regulators were overlapped with CGI coordinates. If at least 20% of the promoter and 20% of the island overlapped, the CGI was assigned as promoter CGI and its mean methylation was used to determine the promoter methylation. If multiple CGIs overlapped a single promoter, the average of all islands was used. Only ARID1B had no promoter CGI assigned and therefore instead of the average methylation within a CGI, the average methylation within the promoter region was computed. For cancer cell lines, CpGs spanned by at least five reads were considered due to some samples with locally poorer coverage.

#### Overrepresentation analysis

Overrepresentation analysis of gene lists in the Gene Ontology term database for biological processes was carried out using the R package (and function) WebGestaltR (parameters: minNum, 10; maxNum, 500; sigMethod, ‘top’; and topThr, 20)^76^.

#### Oncoprint

Mutations across patients with T-ALL were visualized using the R package ComplexHeatmap.

#### Survival analysis

Event-free survival and overall survival from diagnosis were estimated using the Kaplan–Meier method. All groups were compared using the log-rank test^77^. An event was defined as a failure to achieve remission, a relapse after remission or the development of a second malignancy. Analyses were performed using Prism software, v.8.0 (GraphPad Software) and SAS software, v.9.1.2 (SAS Institute).

### Statistics and reproducibility

No statistical method was used to predetermine sample size and maximal number of cases were included based on sample and data availability, which is the goal of providing representation of different T-ALL and B-ALL subtypes and capacity of the WGBS pipeline in the Pediatric Cancer Genome Project. The number of ALL cases included in the current study is much greater than previous publications investigating DNA methylation in ALL and we indicated in the manuscript that the sample size was not large enough for specific analyses such as association with clinical outcome. No data were excluded from the analyses. Randomization and blinding were not relevant for this study as this was not an intervention study. All statistical tests were two-sided and were chosen as appropriate for data distribution. The threshold for statistical significance was defined as *P* < 0.01 or *P* < 0.05 for FDR-corrected *P* values. To explore the role of TET2, a single clonal KO line was generated in Jurkat cells. No replicates were generated. Details of reproducibility are included in the Reporting Summary.

### Reporting Summary

Further information on research design is available in the Nature Research Reporting Summary linked to this article.

## Supplementary information

Supplementary Information Supplementary Fig. 1

Reporting Summary

Supplementary Tables Supplementary Tables 1–16

## Data Availability

WGBS data of primary ALL and healthy samples as patient-derived have been deposited in the European Genome–Phenome archive (accession no. EGAS00001005203). WGBS of B-ALL and T-ALL cell lines as well as RNA-seq data of the T-ALL cell lines DND41 and Jurkat have been deposited in the Gene Expression Omnibus under accession no. GSE164040. RNA-seq and DNA-seq datasets of primary ALL and healthy samples have been obtained from or uploaded to EGAS00001005203, EGAS00001004810, EGAS00001005250, EGAS00001005084, EGAS00001001923, EGAS00001003266, EGAS00001000654 and phs000218 (dbGaP) as listed in Supplementary Table 1. Previously published data from the Blueprint epigenome project that were re-analyzed here were obtained from http://dcc.blueprint-epigenome.eu/ and sample IDs are listed in Supplementary Table 3. Chromatin states of HSCs (Roadmap Epigenome ID E035) and DND41 (Roadmap Epigenome ID E115) were downloaded from the Roadmap Epigenomics Consortium (https://egg2.wustl.edu/roadmap/web_portal/chr_state_learning.html). The human solid tumor data (BLCA, BRCA, COAD, LUAD, LUSC, STAD, READ and UCEC) were derived from the TCGA Research Network: http://cancergenome.nih.gov/ and the corresponding methylation rates and coverage information were downloaded from https://zwdzwd.github.io/pmd. Source data have been provided as Source Data files and source data for all figures have been deposited at https://zenodo.org/record/6337435#.Yk7ExjXTVpg. All other data supporting the findings of this study are available from the corresponding author on reasonable request. Source data are provided with this paper.

## Source data

Source Data Extended Data Fig. 7 Unprocessed gel Extended Data Fig. 7fp.

## Code availability

Code is available at https://github.com/sarahet/The_Distinct_DNA_Methylome_ALL.
