# Supplementary material for: Acute lymphoblastic leukemia displays a distinct highly methylated genome
Source: Nat Cancer. 2022 May 19;3(6):768–82. doi: 10.1038/s43018-022-00370-5 (PMC9236905; doi:10.1038/s43018-022-00370-5)
Supplement: Supplementary file 1 — Supplementary Fig. 1 [file 43018_2022_370_MOESM1_ESM.pdf]

nature portfolio

https://doi.org/10.1038/s43018-022-00370-5

Supplementary information

Acute lymphoblastic leukemia displays a distinct highly methylated genome

In the format provided by the authors and unedited

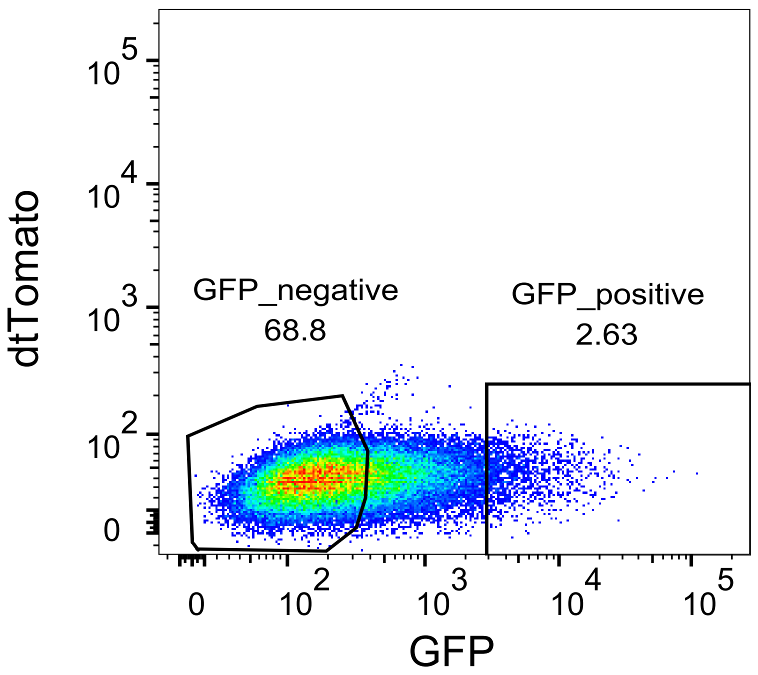

Supplementary Figure 1: Gating strategy for sorting Jurkat TET2 KO candidates. Percentages of sorted cells were analysed using FlowJo. FSC-A/SSC-A was used to determine live cells, followed by gating single cells using FSC-A/FSC-W and SSC-A/SSC-W. Finally, candidates with a high Cas9-GFP signal were sorted.
