# Supplementary material for: Acute lymphoblastic leukemia displays a distinct highly methylated genome
Source: Nat Cancer. 2022 May 19;3(6):768–82. doi: 10.1038/s43018-022-00370-5 (PMC9236905; doi:10.1038/s43018-022-00370-5)
Supplement: Supplementary file 2 — Reporting Summary [file 43018_2022_370_MOESM2_ESM.pdf]

nature research

Corresponding author(s): Alexander Meissner, Charles Mullighan

Last updated by author(s): Mar 10, 2022

# Reporting Summary

Nature Research wishes to improve the reproducibility of the work that we publish. This form provides structure for consistency and transparency in reporting. For further information on Nature Research policies, see our Editorial Policies and the Editorial Policy Checklist.

## Statistics

For all statistical analyses, confirm that the following items are present in the figure legend, table legend, main text, or Methods section.

| n/a | Confirmed | |
|---|---|---|
| ☐ | ☒ | The exact sample size (*n*) for each experimental group/condition, given as a discrete number and unit of measurement |
| ☐ | ☒ | A statement on whether measurements were taken from distinct samples or whether the same sample was measured repeatedly |
| ☐ | ☒ | The statistical test(s) used AND whether they are one- or two-sided<br>*Only common tests should be described solely by name; describe more complex techniques in the Methods section.* |
| ☐ | ☒ | A description of all covariates tested |
| ☐ | ☒ | A description of any assumptions or corrections, such as tests of normality and adjustment for multiple comparisons |
| ☐ | ☒ | A full description of the statistical parameters including central tendency (e.g. means) or other basic estimates (e.g. regression coefficient) AND variation (e.g. standard deviation) or associated estimates of uncertainty (e.g. confidence intervals) |
| ☐ | ☒ | For null hypothesis testing, the test statistic (e.g. $F$, $t$, $r$) with confidence intervals, effect sizes, degrees of freedom and $P$ value noted<br>*Give P values as exact values whenever suitable.* |
| ☒ | ☐ | For Bayesian analysis, information on the choice of priors and Markov chain Monte Carlo settings |
| ☒ | ☐ | For hierarchical and complex designs, identification of the appropriate level for tests and full reporting of outcomes |
| ☐ | ☒ | Estimates of effect sizes (e.g. Cohen's *d*, Pearson's *r*), indicating how they were calculated |

*Our web collection on statistics for biologists contains articles on many of the points above.*

## Software and code

Policy information about availability of computer code

| | |
|---|---|
| Data collection | trimgalore (version 0.4.4), cutadapt (version 2.4), BSMAP (version 2.90), GATK (version 4.1.4.1), MOABS (version 1.3.2), STAR (version 2.7.5a), stringtie (version 2.0.6), FlowJo (version: 10.3) |
| Data analysis | R (version 3.6.3), bedtools (version 2.29.2), pheatmap (version 1.0.12), ComplexHeatmap (2.5.3), metilene (version 0.2-8), DESeq2 (version 1.26.0), ConsensusClusterPlus (version 1.48.0), RLM (v.1.0.0), GraphPad Prism (version 8.0), SAS software (version 9.1.2)<br>Code has been deposited at https://github.com/sarahet/The_Distinct_DNA_Methylome_ALL |

For manuscripts utilizing custom algorithms or software that are central to the research but not yet described in published literature, software must be made available to editors and reviewers. We strongly encourage code deposition in a community repository (e.g. GitHub). See the Nature Research guidelines for submitting code & software for further information.

## Data

Policy information about availability of data

All manuscripts must include a data availability statement. This statement should provide the following information, where applicable:

- Accession codes, unique identifiers, or web links for publicly available datasets
- A list of figures that have associated raw data
- A description of any restrictions on data availability

WGBS data of primary ALL and normal samples as patient derived have been deposited in the European Genome Phenome archive (accession EGAS00001005203).
WGBS of B-ALL and T-ALL cell lines as well as RNAseq data of the T-ALL cell lines DND41 and Jurkat have been deposited in the Gene Expression Omnibus under accession GSE164040.
RNAseq and DNAseq data sets of primary ALL and normal samples have been obtained from or uploaded to EGAS00001005203, EGAS00001004810, EGAS00001005250, EGAS00001005084, EGAS00001001923, EGAS00001003266, EGAS00001000654 and phs000218 (dbGaP) as listed in Supplementary Table 1.

Previously published data from the Blueprint epigenome project that were re-analyzed here were obtained from http://dcc.blueprint-epigenome.eu/ and sample IDs are listed in Supplementary Table 2. Chromatin states of hematopoietic stem cells (Roadmap Epigenome ID: E035) and DND41 (Roadmap Epigenome ID: E115) were downloaded from the Roadmap Epigenomics Consortium (https://egg2.wustl.edu/roadmap/web_portal/chr_state_learning.html). The human solid tumor data (BLCA, BRCA, COAD, LUAD, LUSC, STAD, READ and UCEC) were derived from the TCGA Research Network: http://cancergenome.nih.gov/ and the corresponding methylation rates and coverage information were downloaded from https://zwdzwd.github.io/pmd.
Source data have been provided as Source Data files, and source data for all figure panels has been deposited at 10.5281/zenodo.6337435. All other data supporting the findings of this study are available from the corresponding author on reasonable request.

# Field-specific reporting

Please select the one below that is the best fit for your research. If you are not sure, read the appropriate sections before making your selection.

☒ Life sciences ☐ Behavioural & social sciences ☐ Ecological, evolutionary & environmental sciences

For a reference copy of the document with all sections, see nature.com/documents/nr-reporting-summary-flat.pdf

# Life sciences study design

All studies must disclose on these points even when the disclosure is negative.

| | |
|---|---|
| Sample size | Samples were selected based on sample availability, availability of prior genomic data, the goal of providing representation of different T-ALL and B-ALL subtypes, and capacity of the WGBS pipeline in the Pediatric Cancer Genome Project. We carefully controlled for false discoveries by using stringent significance thresholds and indicated in the manuscript that the sample size was not large enough for specific analyses such as association with clinical outcome. Sample sizes are indicated in the figure panels or legends. |
| Data exclusions | No data was excluded. |
| Replication | To explore the role of TET2, a single clonal knockout line was generated in JURKAT cells. No replicates were generated. |
| Randomization | Not relevant to this study, since this is not an intervention study. |
| Blinding | Blinding was not relevant for this study since this is not an intervention study. However, our analytical pipeline followed uniform criteria applied to all samples, allowing us to analyze our data in an unbiased manner. |

# Reporting for specific materials, systems and methods

We require information from authors about some types of materials, experimental systems and methods used in many studies. Here, indicate whether each material, system or method listed is relevant to your study. If you are not sure if a list item applies to your research, read the appropriate section before selecting a response.

## Materials & experimental systems

| n/a | Involved in the study | |
|---|---|---|
| ☒ | ☐ | Antibodies |
| ☐ | ☒ | Eukaryotic cell lines |
| ☒ | ☐ | Palaeontology and archaeology |
| ☒ | ☐ | Animals and other organisms |
| ☐ | ☒ | Human research participants |
| ☒ | ☐ | Clinical data |
| ☒ | ☐ | Dual use research of concern |

## Methods

| n/a | Involved in the study | |
|---|---|---|
| ☒ | ☐ | ChIP-seq |
| ☐ | ☒ | Flow cytometry |
| ☒ | ☐ | MRI-based neuroimaging |

## Eukaryotic cell lines

Policy information about cell lines

| | |
|---|---|
| Cell line source(s) | Jurkat (ACC 282, DSMZ), DND41 (ACC 525, DSMZ), PEER (ACC6, DSMZ), PER-117 (Gift from Ursula Kees, Perth), MOLT-16 (ACC29, DSMZ), RPMI-8402 (ACC290, DSMZ), LOUCY (ACC394, DSMZ), TALL-1 (ACC521, DSMZ), ALL-SIL (ACC511, DSMZ), NALM-6 (ACC128, DSMZ), NALM-16 (ACC680, DSMZ), MHH-CALL-2 (ACC341, DSMZ), MHH-CALL-4 (ACC337, DSMZ), MUTZ5 (ACC490, DSMZ) |
| Authentication | No authentification of DND-41 and Jurkat beyond the information provided by the DSMZ. Identification for other ALL cell lines was verified by STR genotyping. |
| Mycoplasma contamination | All cell lines were tested for mycoplasma and were found to be mycoplasma negative. |
| Commonly misidentified lines (See ICLAC register) | No commonly misidentified cell lines were used. |

## Human research participants

Policy information about studies involving human research participants

| | |
|---|---|
| Population characteristics | Leukemia and normal samples were studied from patients with ALL (T-ALL, DUX4/ERG B-ALL, hypodiploid B-ALL or Ph-like B-ALL). Patients involved both children and adults, male and female. Exact details of demographic data of each participant are included in the Methods and Supplementary Table 1. |
| Recruitment | Patients with ALL were included in this study primarily through the participation in therapeutic trials and/or tissue banking protocols. Patients were not specifically recruited for the purpose of this research. Individuals were included in various analyses of this study based on sample availability. We are not aware of any bias in recruitment and sampling and we do not believe our results are impacted by any sampling bias. Samples were de-identified, with no interaction between the investigators and the research subjects. Patients and/or guardians provided informed consent/assent. |
| Ethics oversight | Research was approved by the St Jude IRB as being "non human subjects research". |

Note that full information on the approval of the study protocol must also be provided in the manuscript.

## Flow Cytometry

### Plots

Confirm that:

☒ The axis labels state the marker and fluorochrome used (e.g. CD4-FITC).

☒ The axis scales are clearly visible. Include numbers along axes only for bottom left plot of group (a 'group' is an analysis of identical markers).

☒ All plots are contour plots with outliers or pseudocolor plots.

☒ A numerical value for number of cells or percentage (with statistics) is provided.

### Methodology

| | |
|---|---|
| Sample preparation | JURKAT cells were washed in PBS, centrifuged, and resuspended in PBS+1% FBS for sorting |
| Instrument | BD FACSAriaII (BD, San Jose, CA, USA) |
| Software | FlowJo |
| Cell population abundance | Cells containing the highest GFP signal representing about 5% of all cells were sorted as single cells into a 96 well plate where they were clonally propagated and genotyped using their gDNA and cDNA. Purity was assessed based on a sort check and then the subsequent genotype. |
| Gating strategy | WT untransfected JURKAT cells were analyzed first to set the gating for the negative control. Next, JURKAT cells transfected with the px458 plasmid containing our gRNA and a GFP reporter were analyzed and the GFP+ gate was drawn around the fraction of cells with the highest expression. |

☒ Tick this box to confirm that a figure exemplifying the gating strategy is provided in the Supplementary Information.
