# Supplementary figures and images for: Acute lymphoblastic leukemia displays a distinct highly methylated genome
Source: Nat Cancer. 2022 May 19;3(6):768–82. doi: 10.1038/s43018-022-00370-5 (PMC9236905; doi:10.1038/s43018-022-00370-5)

**a** Unprocessed image matching Extended Data Fig. 7f

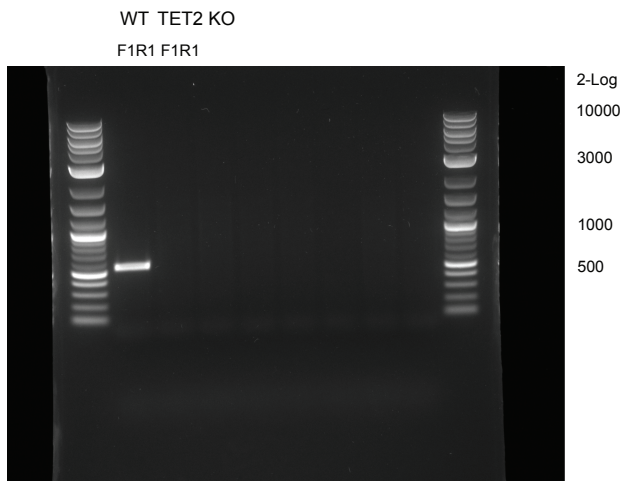

Supplement: Source Data Extended Data Fig. 7 — Unprocessed gel Extended Data Fig. 7fp. [file 43018_2022_370_MOESM4_ESM.pdf]
